# Selective Duplex Formation in Mixed Sequence Libraries of Synthetic Polymers

**DOI:** 10.1021/jacs.4c01381

**Published:** 2024-03-26

**Authors:** Mohit Dhiman, Ronan Cons, Oliver N. Evans, Joseph T. Smith, Cecilia J. Anderson, Rafel Cabot, Daniil O. Soloviev, Christopher A. Hunter

**Affiliations:** Yusuf Hamied Department of Chemistry, University of Cambridge, Lensfield Road, Cambridge CB2 1EW, U.K.

## Abstract

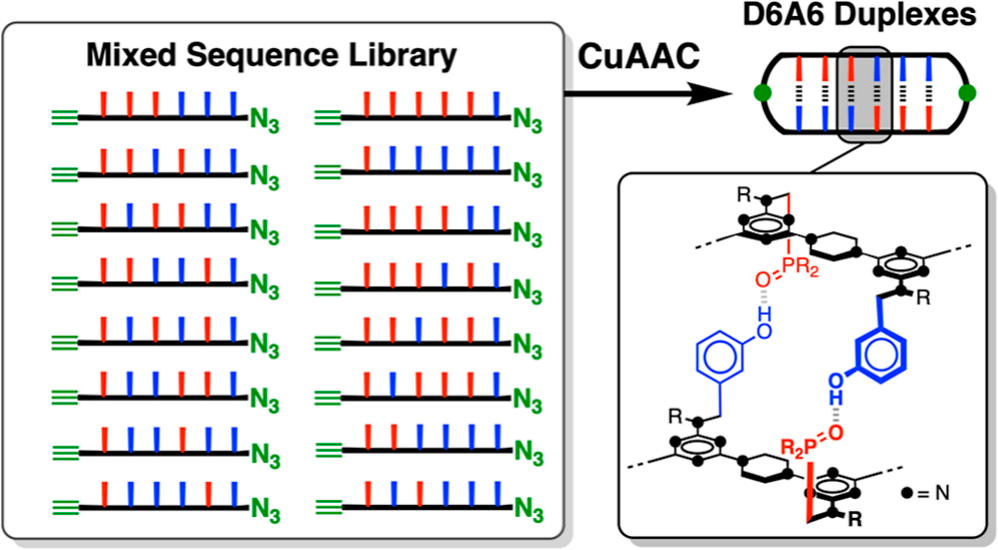

Recognition-encoded melamine oligomers (REMO) are synthetic polymers that feature an alternating 1,3,5-triazine-piperazine backbone and side-chains equipped with either a phenol or phosphine oxide recognition unit. An automated method for the solid-phase synthesis (SPS) of REMO of any specified sequence has been developed starting from dichlorotriazine monomer building blocks. Complementary homo-oligomers with either six phenols or six phosphine oxides were synthesized and shown to form a stable duplex in nonpolar solvents by NMR denaturation experiments. The duplex was covalently trapped by equipping the ends of the oligomers with an azide and an alkyne group and using a copper-catalyzed alkyne–azide cycloaddition (CuAAC) reaction. The SPS methodology was adapted to synthesize mixed sequence libraries by using a mixture of two different dichlorotriazine building blocks in each coupling cycle of an oligomer synthesis. The resulting libraries contain statistical mixtures of all possible sequences. The self-assembly properties of these libraries were screened by using the CuAAC reaction to trap any duplexes present. In mixed sequence libraries of 6-mers, the trapping experiments showed that only sequence-complementary oligomers formed duplexes at micromolar concentrations in dichloromethane. The automated synthesis approach developed here provides access to large libraries of mixed sequence synthetic polymers, and the covalent trapping experiment provides a convenient tool for screening functional properties of mixtures. The results suggest high-fidelity sequence-selective duplex formation in mixtures of 6-mer sequences of the REMO architecture.

## Introduction

The most striking feature of the molecular machinery found in nature is the simplicity of the chemical structures of biomolecules that are responsible for recognition, catalysis, and self-assembly. The key compounds are almost exclusively linear polymers, where function is programmed using the sequence of monomer building blocks. It should be possible to encode function in the same way in synthetic polymers made from two or more different monomer units, but such materials remain a relatively unexplored area of chemistry, because both synthesis and characterization are challenging.

**Figure 1:**
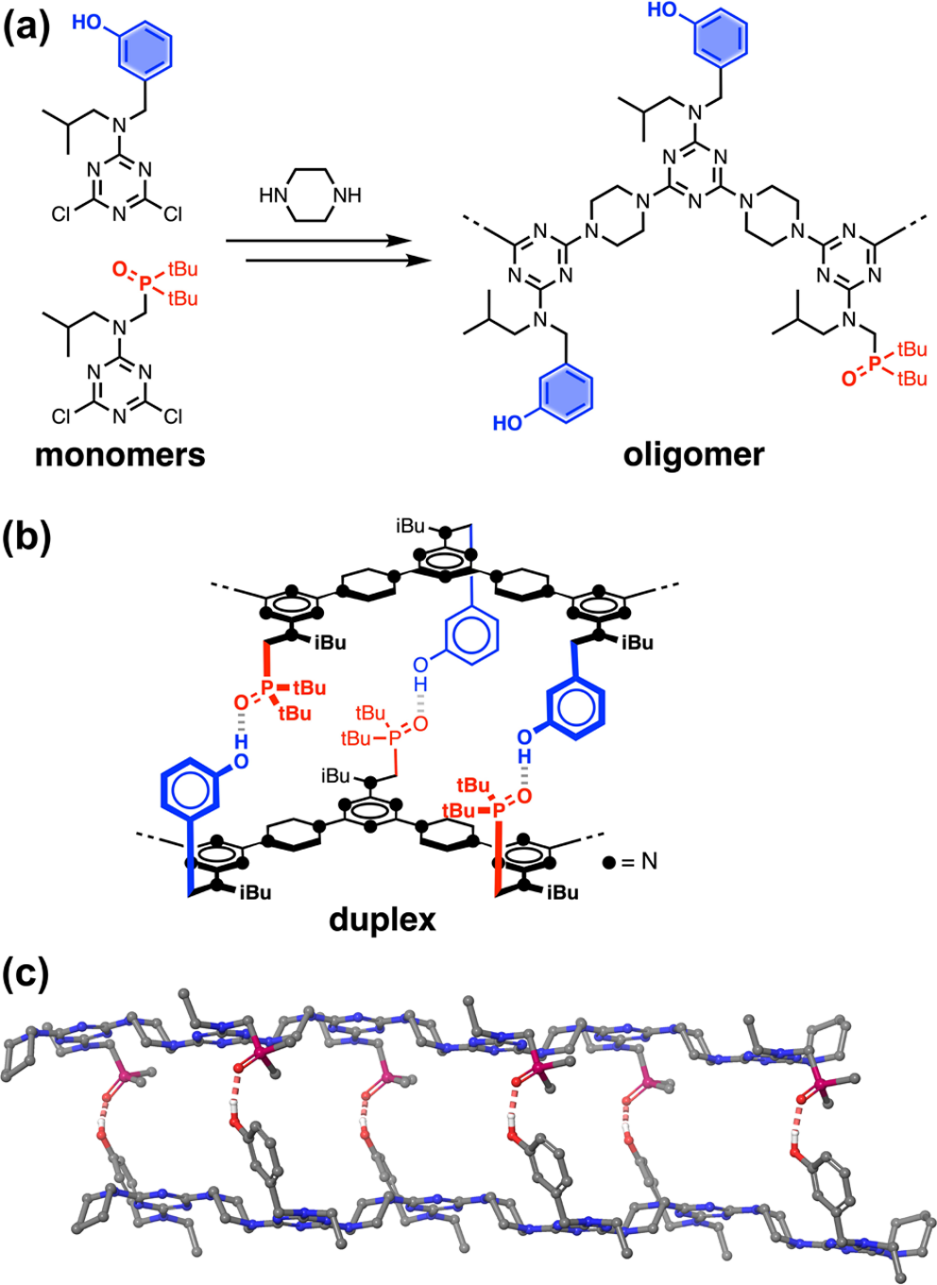
REMO. (a) Oligomers are synthesized from dichlorotriazines equipped with complementary recognition units using S_N_Ar reactions with piperazine. (b) Phenol•phosphine oxide H-bonding interactions lead to the assembly of duplexes between complementary sequences. (c) Molecular mechanics model of a duplex formed between a phosphine oxide 6-mer and a phenol 6-mer (energy minimization using a customized OPLS4 force-field with chloroform solvation in Macromodel).^13^ The *i*-butyl and *t*-butyl groups have been truncated to methyl groups for the sake of clarity.

Drawing inspiration from the ladder motif in the DNA double helix, synthetic chemists have developed short oligomers that form duplexes using different kinds of noncovalent interaction.^1−11^ We recently reported one such system, recognition-encoded melamine oligomers (REMO), which have a uniform backbone composed of alternating piperazine and triazine units and a sequence defined by side-chains that carry either a phenol or a phosphine oxide group (Figure 1a). H-bonding interactions between complementary side-chains led to sequence-selective duplex formation of 3-mers in toluene solution (Figure 1b).^12^ Here we establish a general synthetic approach to REMO and show that high-fidelity duplex formation persists in longer mixed sequence polymers (Figure 1c).

A major challenge in studying synthetic sequence polymers is that the number of possible compounds increases exponentially with the chain length. For example, if the two different monomer units shown in Figure 1a are used, 36 different 6-mers are possible, which is a challenge for synthesis, and characterization of the selectivity of duplex formation would require measurements of over 1000 different pairwise interactions. Progress in this field therefore requires the development of efficient methods for the synthesis of libraries of sequences and experimental approaches that probe the functional properties of large numbers of different compounds in a highly parallel manner. Automated solid-phase synthesis (SPS) offers an attractive solution to this synthesis problem. The REMO architecture is an ideal target for SPS methods, because the S_N_Ar coupling reactions used to build the oligomers are particularly high yielding, produce no significant side products, and require no reagents other than a base.^14^ In this paper, we introduce an automated SPS method that gives access to REMO of a specific chain length and sequence. SPS was used to create libraries of 6-mer sequences, and a method was developed for screening the self-assembly properties of the mixtures using copper-catalyzed azide–alkyne cycloaddition (CuAAC) reactions to covalently trap duplexes formed by complementary pairs of sequences. The results indicate that the REMO architecture provides a robust platform for the development of synthetic polymers that can process chemical information in a manner analogous to that of nucleic acids.

## Results and Discussion

### Automated SPS

**Figure 2:**
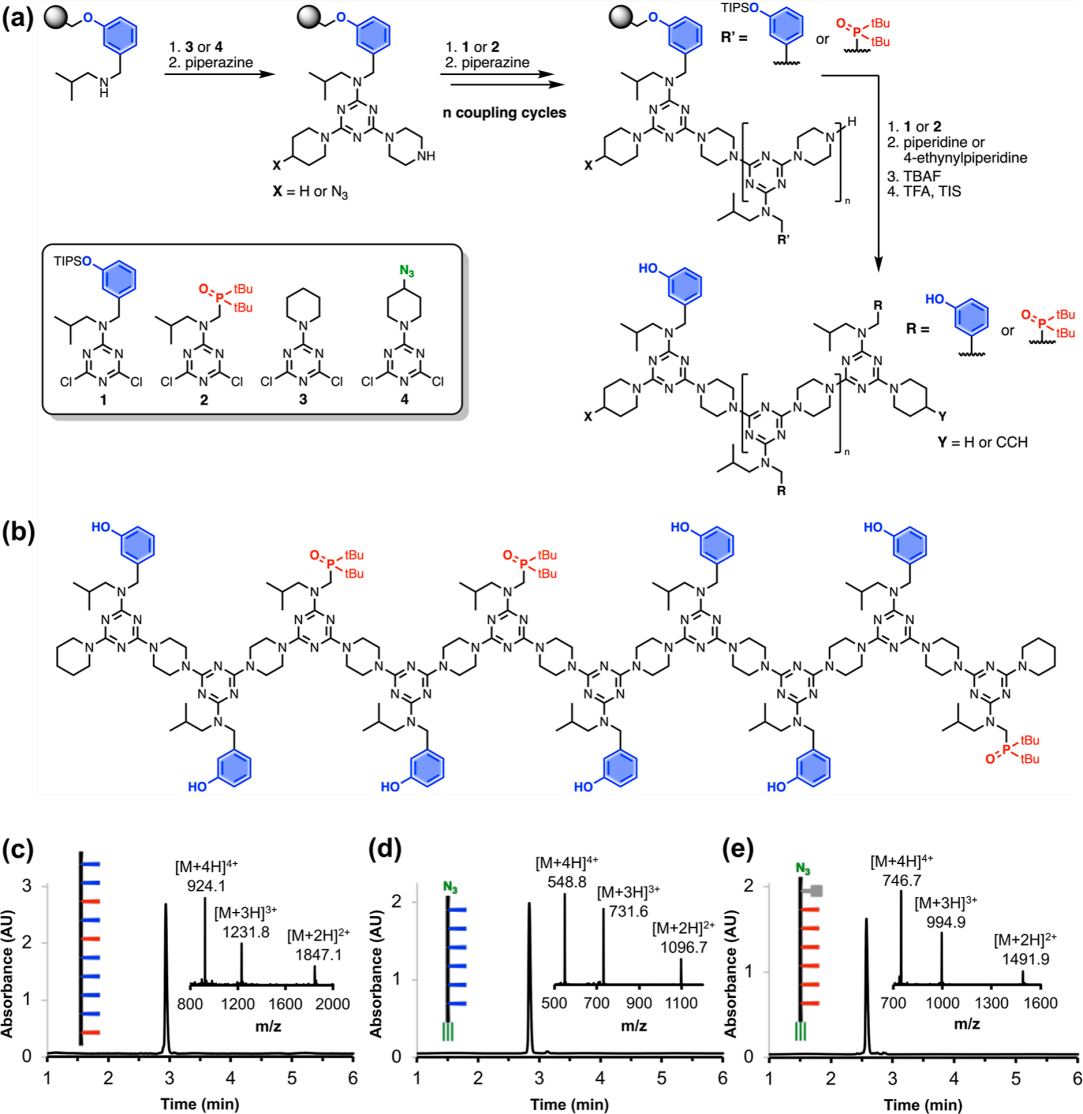
Automated SPS of the REMO. (a) SPS protocols for REMO synthesis and dichlorotriazine building blocks **1**–**4**. (b) Structure of **pDDADADDDDAp**. (c) UPLC trace and ESI-MS of the crude product, **pDDADADDDDAp**, and cartoon representation of the structure. Calculated mass (ESI^+^): 1847.2 [M + 2H]^2+^, 1231.7 [M + 3H]^3+^, 924.2 [M + 4H]^4+^. (d) UPLC trace and ESI-MS of crude product, **zDDDDDDy**, and cartoon representation of the structure showing the terminal alkyne and azide groups in green. Calculated mass (ESI^+^): 1096.7 [M + 2H]^2+^, 731.4 [M + 3H]^3+^, 548.8 [M + 4H]^4+^. (e) UPLC trace and ESI-MS of crude product, zD*AAAAAAy. The cartoon representation of the structure shows the acetylated phenol unit in gray. Calculated mass (ESI^+^): 1492.0 [M + 2H]^2+^, 995.0 [M + 3H]^3+^, and 746.5 [M+4H]^4+^. UPLC conditions: C4 column at 40 °C using a 30–100% gradient of THF/formic acid (0.1%) in water/formic acid (0.1%) over 4 min, then 100% THF/formic acid (0.1%) over 2 min.

SPS was originally developed for the synthesis of peptides and oligonucleotides and has had a major impact on our understanding of the relationship between the sequence and properties of biopolymers.^15,16^ A number of different kinds of synthetic polymer have been made using SPS,^17−26^ and Huc has shown that it is possible to use the sequence of building blocks to control the folding and recognition properties of aromatic amide oligomers.^27^ The development of an SPS route for REMO synthesis is illustrated in Figure 2a. The key reagents are dichlorotriazines, which can be prepared in multigram quantities from cyanuric chloride and the relevant amine.^12,22^ Dichlorotriazines **1** and **2** are equipped with either a phenol or a phosphine oxide recognition unit, and dichlorotriazines **3** and **4** were used to cap the chain ends or introduce a terminal azide group. TentaGel Wang resin was functionalized with a phenol recognition unit equipped with a secondary amine (see Supporting Information), and oligomers were assembled by iterative rounds of S_N_Ar reactions, alternately coupling with one of the dichlorotriazines then piperazine (Figure 2a). In the final step, the oligomer was capped with either piperidine or 4-ethynylpiperidine, before the deprotection of the phenol groups and cleavage from the resin.

The iterative rounds of coupling were automated using a CEM Liberty Blue peptide synthesizer, which enabled synthesis of REMO at a rate of two melamine units per hour, and high purity polymers were obtained directly on cleavage from the resin. The sequences of the oligomers are described using upper case letters for the recognition units (**D** for phenol, D* for acetylated phenol, **A** for phosphine oxide, and **X** for an undefined recognition unit in a mixture) and lower case letters for the end groups (**p** for piperidine, **z** for azide, and **y** for alkyne). Figure 2b shows the structure of a mixed sequence 10-mer **pDDADADDDDAp** that was synthesized by automated SPS, and Figure 2c shows the UPLC trace and ESI-MS of the crude product. The development of this robust automated synthesis methodology opens the way for exploring the relationship between sequence and properties, and here, we investigate the sequence selectivity of duplex formation using 6-mers.

Two homo-oligomers equipped with terminal alkyne and azide groups were first synthesized in order to develop methods for characterization of a H-bonded REMO duplex. The route shown in Figure 2a uses a terminal phenol unit to grow oligomers on the resin, which means that the first recognition unit in the sequence is always a phenol. Therefore, **zDDDDDDy** was obtained directly by automated SPS, but a modification was required to obtain the complementary homo-oligomer containing six phosphine oxides. This oligomer was accessed by first synthesizing **zDAAAAAAy** using automated SPS, and then acetylating the terminal phenol group to prevent it from acting as a H-bond donor. The resulting acetate ester is a weak H-bond acceptor (H-bond acceptor parameter β = 5) and cannot compete with the phosphine oxide recognition units (β = 11) for H-bonding interactions with the phenol oligomer.^28−30^Figure 2d shows the UPLC trace and ESI-MS of the crude **zDDDDDDy** product obtained from the synthesizer, and Figure 2e shows the corresponding data for the zD*AAAAAAy product obtained after acetylation of the oligomer made by automated SPS.

### Library Synthesis

In addition to synthesizing discrete oligomers of specified sequence, the automated SPS methodology can be used to make libraries containing mixtures of different sequences. If a mixture of two different dichlorotriazines is added in one of the coupling cycles in Figure 2a, then either of the two different building blocks could be incorporated into the growing chain, and the resulting product will be a mixture of oligomers with two different sequences. If the dichlorotriazines have identical reactivity, then they will be incorporated with equal probability, but small differences in reactivity can be compensated for by changing the ratio of the two reactants. We found that a 3:2 mixture of **1** and **2** can be used to ensure that an equal proportion of the two recognition units are incorporated in a coupling cycle (see Supporting Information for details). If this mixture of **1** and **2** were used in every cycle of SPS, the result would be a library of all possible sequences that start with a **D**.

**Figure 3:**
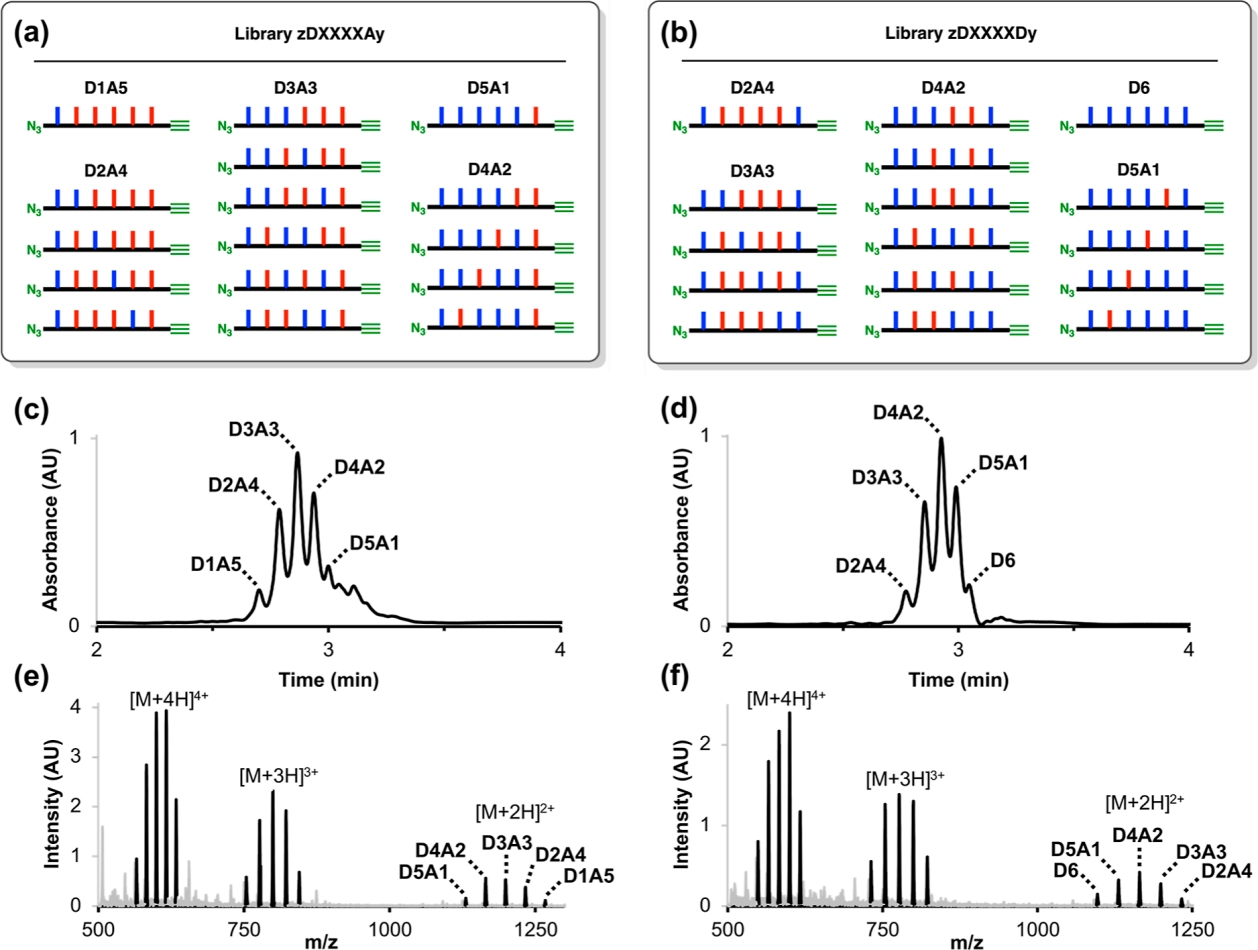
Mixed sequence 6-mer libraries. Schematic representation of the sequences present in (a) library **zDXXXXAy** and (b) library **zDXXXXDy**. UPLC traces of crude products, (c) library **zDXXXXAy** and (d) library **zDXXXXDy**, with peaks labeled according to the composition of recognition units. ESI-MS of crude products, (e) library **zDXXXXAy** and (f) library **zDXXXXDy**. The [M + 2H]^2+^ peaks are labeled according to the composition of recognition units, and the corresponding [M + 3H]^3+^ and [M + 4H]^4+^ peaks are highlighted in black. UPLC conditions: C4 column at 40 °C using a 30–100% gradient of THF/formic acid (0.1%) in water/formic acid (0.1%) over 4 min, then 100% THF/formic acid (0.1%) over 2 min.

This approach was used to synthesize two different mixed sequence 6-mer libraries in order to investigate the sequence selectivity of duplex formation. Oligomers in library **zDXXXXAy** begin with a phenol, followed by all possible sequences of the next four recognition units, and end with a terminal phosphine oxide (Figure 3a). Oligomers in library **zDXXXXDy** begin with a phenol, followed by all possible sequences of the next four recognition units, and end with a terminal phenol (Figure 3b). Thus, in library **zDXXXXAy**, each oligomer has a complementary partner with which it can form a duplex with six H-bonds, whereas there are no mutually complementary sequences in library **zDXXXXDy**.

Figure 3c,d show the UPLC traces of the crude libraries obtained by automated SPS. In both libraries, there were five clearly resolved peaks, which could be assigned to oligomers with different numbers of phenol and phosphine oxide groups by mass spectrometry. The UPLC retention time depends on the composition of the oligomer, but for oligomers with the same composition, individual sequences could not be resolved. Figure 3e,f show the ESI-MS of the two libraries with signals labeled according to the composition of the oligomer (see Table S1 for calculated and found masses). The areas of the UPLC peaks and the intensities of the ESI-MS signals both indicate a binomial distribution of the five possible oligomer compositions. This result confirms that each step in the coupling cycle incorporated equal amounts of the two recognition units, and we conclude that a statistical distribution of all 16 sequences shown in Figure 3a,b is present in each library. The UPLC trace of library **zDXXXXAy** indicates that some impurities were present (retention times 3.0–3.2 min), but the crude mixtures were used in the duplex screening experiments described below without any further purification.

### Duplex Formation by Homo-Oligomers

Homo-oligomer **zDDDDDDy** proved difficult to dissolve in nonpolar solvents, but addition of zD*AAAAAAy dramatically increased the solubility, which suggests that the two oligomers form a soluble complex. A 1 mM solution of a 1:1 mixture of **zDDDDDDy** and zD*AAAAAAy in 1,1,2,2-tetrachloroethane-*d*_2_ (TCE-*d*_2_) was used to investigate duplex formation by NMR spectroscopy. In the ^31^P NMR spectrum of the mixture, the signals due to the zD*AAAAAAy phosphine oxide groups appeared as a single broad peak at 61.5 ppm, which is a 2.4 ppm downfield shift compared with a sample of pure zD*AAAAAAy. The large increase in chemical shift observed in the mixture is characteristic of H-bonding interactions between the phosphine oxides and the complementary phenol recognition units and suggests that all of the phosphine oxide groups are involved in intermolecular base-pairing interactions with phenols in the zD*AAAAAAy•**zDDDDDDy** duplex.^11^ On heating the mixture of oligomers, the chemical shift of the signal due to the phosphine oxide groups decreased to eventually reach the same chemical shift as the signal observed for pure zD*AAAAAAy at 363 K (Figure S23), which is consistent with denaturation of the duplex to give the two single strands at high temperatures.

**Figure 4:**
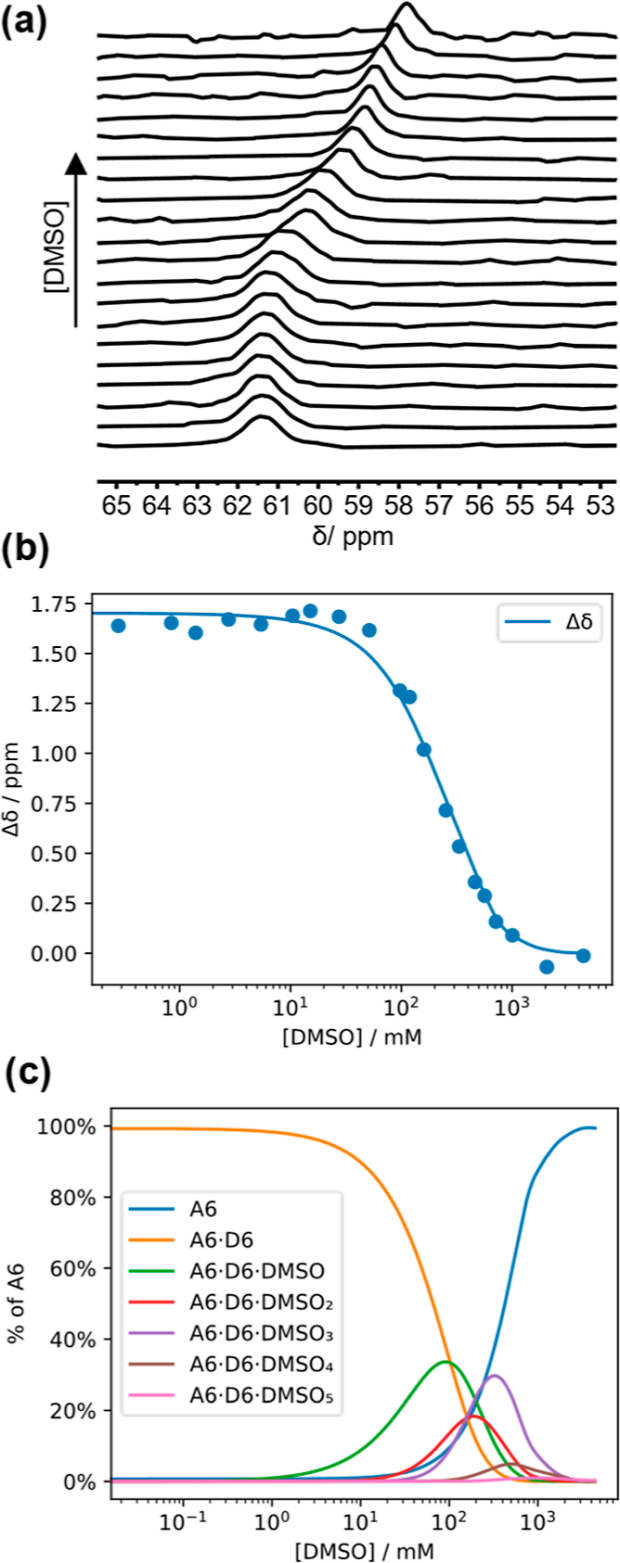
DMSO denaturation of the zD*AAAAAAy•**zDDDDDDy** duplex. (a) ^31^P NMR spectra (162 MHz) for titration of DMSO-*d*_6_ (0–4 M) into a 1 mM 1:1 mixture of **zDDDDDDy** and zD*AAAAAAy in TCE-*d*_2_ at 298 K. (b) Complexation-induced change in ^31^P NMR chemical shift (Δδ) for duplex denaturation plotted as a function of DMSO-*d*_6_ concentration in TCE-*d*_2_ at 298 K (Δδ is the difference between the chemical shift of the 1:1 mixture and pure zD*AAAAAAy at the same concentration of DMSO). The line was calculated using a denaturation isotherm including the partially denatured species listed in (c). (c) Calculated speciation profile plotted as a function of DMSO-*d*_6_ concentration in TCE-*d*_2_ at 298 K (see Figure S26).

Addition of H-bonding competitors like DMSO can also be used to denature H-bonded duplexes.^11^Figure 4a shows ^31^P NMR spectra of a 1:1 mixture of **zDDDDDDy** and zD*AAAAAAy_**6**_ in TCE-*d*_2_ in the presence of increasing amounts of DMSO-*d*_6_. At low concentrations of DMSO-*d*_6_, the signals due to zD*AAAAAAy appeared at 61.5 ppm, characteristic of the H-bonded duplex. At higher concentrations of DMSO-*d*_6_, the chemical shift of the signal due to the phosphine oxide groups decreased to eventually reach the same chemical shift as the signal observed for pure zD*AAAAAAy in 4 M DMSO-*d*_6_. These data were used to determine the association constant for assembly of the duplex (see Supporting Information for details). Although the denaturation data did not fit to a simple two-state, all-or-nothing denaturation isotherm (Figure S25), a good fit was obtained using a model that allowed for the partially denatured intermediates shown in Figure 4c (Figure S26). The association constants for the phenol•phosphine oxide interaction (110 M^–1^) and the phenol•DMSO interaction (17 M^–1^) were independently measured in TCE-*d*_2_. By assuming that the complexation-induced change in chemical shift of each phosphine oxide (Δ∂) is the same and that the effective molarity (EM) for each intramolecular base-pairing interaction in the duplex is the same, it was possible to fit the relatively complicated multistate denaturation isotherm by optimizing just two variables (Figure 4b): Δ∂ = 1.7 ppm and EM = 80 mM. The effective molarity is comparable to the value measured for shorter REMO in toluene (40 mM) and within the range reported for other H-bonded duplexes (10–100 mM).^11^

The results of the fitting process also provide the speciation of all partially denatured intermediates as a function of DMSO concentration (Figure 4c). Each DMSO binding event corresponds to breaking one of the base-pairs in the duplex. Figure 4c shows that as DMSO is added, initially the first few base-pairs dissociate to give significant populations of the partially bound intermediates that bind up to three DMSO molecules, but then complete dissociation of the duplex takes place cooperatively to give the two single strands. The association constant for formation of the zD*AAAAAAy•**zDDDDDDy** duplex is log *K* = 7.2 in TCE-*d*_2_, which is a significant increase in stability compared with previously reported duplexes of short oligomers.^12^

### Covalent Trapping

**Figure 5:**
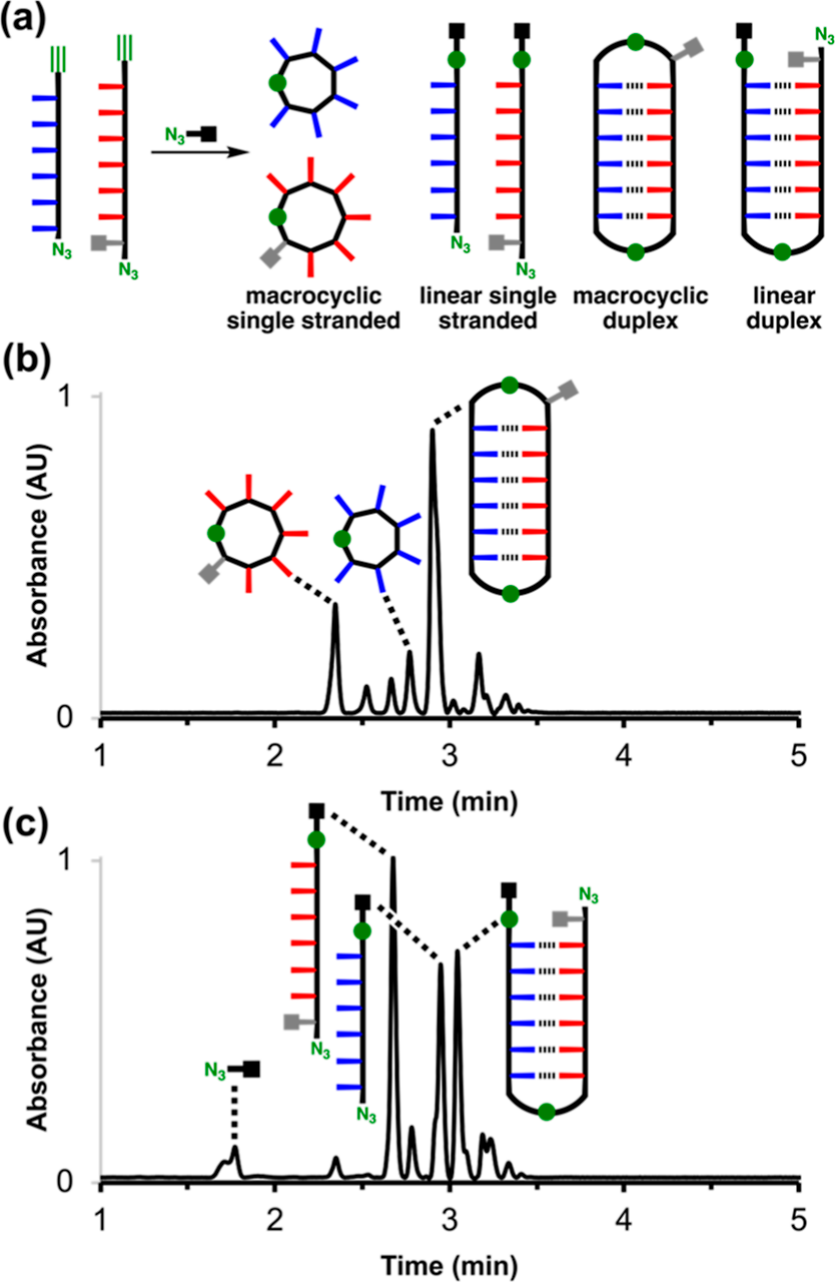
Covalent trapping of the duplex. (a) Schematic representation of products formed after CuAAC reaction of a 1:1 mixture of **zDDDDDDy** and zD*AAAAAAy in the presence of a competing azide (green circles represent triazoles in the products). A linear duplex could also be formed with the azide on the end of the **zDDDDDDy** chain rather than the zD*AAAAAAy chain as drawn. (b) UPLC trace after reaction of **zDDDDDDy** (50 μM), zD*AAAAAAy (50 μM), 4-*t*-butylbenzyl azide (100 μM) and Cu(MeCN)_4_PF_6_-TBTA (0.4 mM) in dichloromethane at room temperature for 48 h. (c) UPLC trace after reaction of **zDDDDDDy** (50 μM), zD*AAAAAAy (50 μM), 4-*t*-butylbenzyl azide (5 mM) and Cu(MeCN)_4_PF_6_-TBTA (0.4 mM) in dichloromethane at room temperature for 48 h. UPLC conditions: C4 column at 40 °C using a 30–100% gradient of THF/formic acid (0.1%) in water/formic acid (0.1%) over 4 min, then 100% THF/formic acid (0.1%) over 2 min.

Both **zDDDDDDy** and zD*AAAAAAy were equipped with terminal alkyne and azide groups, which made it possible to use CuAAC reactions to covalently trap and characterize the supramolecular assemblies present in solution. Figure 5a shows cartoon representations of the products that can be formed when a mixture of zD*AAAAAAy and **zDDDDDDy** is reacted under CuAAC conditions in the presence of another competing azide.^31^ The association constant measured in the denaturation experiment indicates that at a concentration of 50 μM the two oligomers should be fully assembled as the duplex. The macrocyclic duplex product will be formed if the alkyne of one strand reacts with the azide of the other, and then this reaction is repeated at the other end of the duplex. However, if the duplex is sufficiently flexible to fold back on itself, it is also possible for the alkyne of one strand to react with the azide of the same strand, and this process will lead to the macrocyclic single stranded products shown in Figure 5a. Indeed, control experiments carried out with the single stranded zD*AAAAAAy gave quantitative yields of the macrocyclic single stranded product, suggesting that the REMO backbone can readily access conformations that bring the two ends of the oligomer into close proximity (see Figure S27), and this is presumably the case for the duplex as well. In other words, there are two competing intramolecular reaction pathways that can occur within the duplex, and these processes lead to the macrocyclic single stranded and macrocyclic duplex products. When the reaction is carried out in the presence of a competing azide, a third competing process is introduced, an intermolecular reaction with the competing azide, which gives rise to the linear single stranded and linear duplex products in Figure 5a.

Figure 5b shows the UPLC trace after CuAAC reaction of a 1:1 mixture of zD*AAAAAAy and **zDDDDDDy** (50 μM) in the presence of 100 μM of 4-*t*-butylbenzyl azide in dichloromethane. The use of an excess of azide and long reaction times ensured that no unreacted alkynes remained in the final product mixture.^32^ The UPLC peaks were assigned based on the masses observed in the corresponding ESI-MS. The macrocyclic duplex was clearly the major product, which suggests that the duplex is highly populated at μM concentrations, consistent with the association constant measured in the NMR denaturation experiment. Single stranded macrocycles were also observed, but very little of the products due to intermolecular reactions with the competing azide were detected, which indicates that the effective molarities for the intramolecular reactions are all much higher than 100 μM. When the reaction was repeated in the presence of a large excess of 4-*t*-butylbenzyl azide (5 mM), the major products were the linear single strands and the linear duplex (Figure 5c). Under these conditions, intermolecular reactions with the competing azide dominate, which supports the conclusion that formation of the macrocyclic duplex in Figure 5a arises from intramolecular reactions in the duplex. CuAAC trapping therefore provides a useful tool for screening for duplex formation in the more complex mixtures of self-assembled species that are present in mixed sequence REMO libraries. The fact that the linear duplex and single-stranded products are formed in similar amounts in the presence of 5 mM 4-*t*-butylbenzyl azide suggests that the effective molarity for the intramolecular reaction between two different strands in the duplex is around 5 mM. In contrast, effective molarity for the intramolecular process leading to single stranded macrocycles is significantly lower than 5 mM, because these products are completely abolished under these conditions.

### Library Screening

CuAAC reactions were then used to investigate the self-assembly properties of mixed sequence libraries **zDXXXXAy** and **zDXXXXDy** under the same conditions. To ensure approximately 50 μM concentrations of each oligomer in the trapping experiments, a total library concentration of 1 mM was used. Although one equivalent of competing azide was used to intercept intermolecular reactions, the total concentration of alkyne and azide in these experiments was much higher than the experiment shown in Figure 5, so more oligomeric products from intermolecular reactions were observed. Peaks corresponding to macrocyclic single stranded products were clearly resolved in the UPLC trace, but there are multiple overlapping peaks in the region corresponding to macrocyclic and linear duplexes (Figure S28). However, extracted ion chromatograms (EIC) can be used to identify specific products present in the crude reaction mixture and provide a convenient tool to screen for duplex formation by quantifying the amounts of different macrocyclic duplexes formed in the CuAAC reaction.

**Figure 6:**
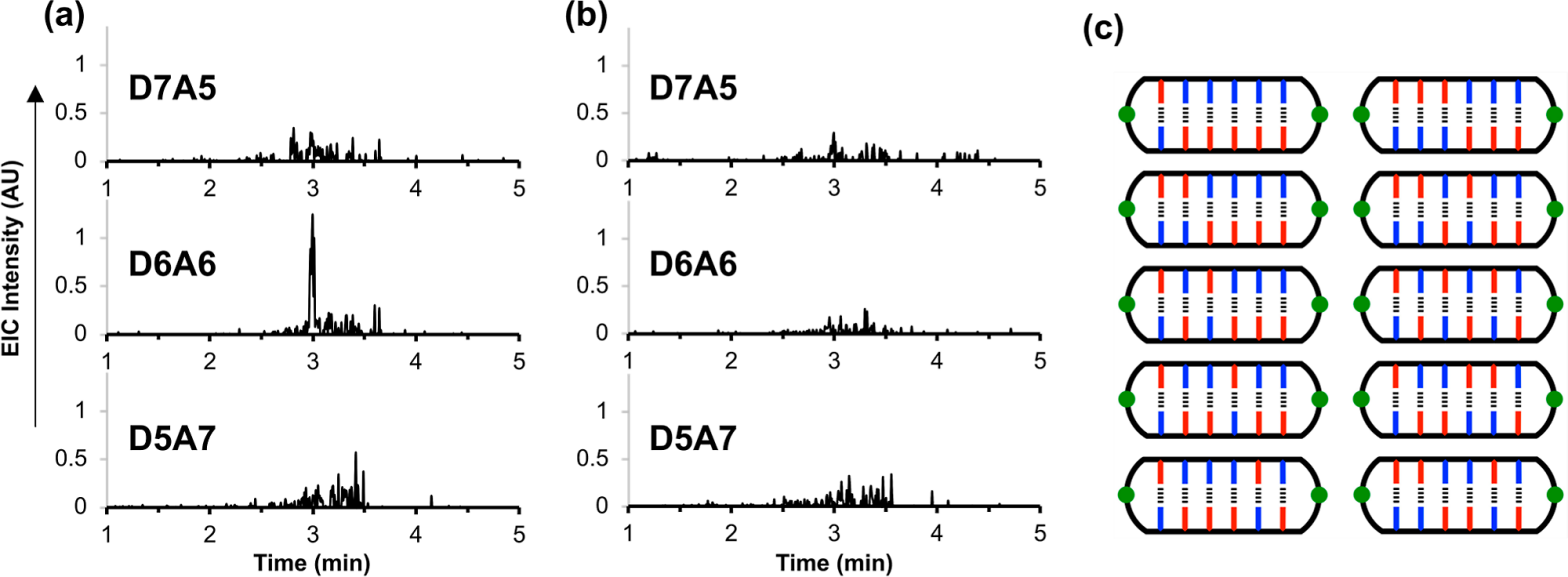
Screening for duplex formation in the mixed sequence libraries. EIC for the [M + 5H]^5+^ ions of macrocyclic duplexes with different numbers of recognition units (see Figure S31 for EIC traces of all possible compositions). (a) Products of the CuAAC reaction of library **zDXXXXAy**; (b) products of the CuAAC reaction of library **zDXXXXDy**. Reactions were carried out using 1 mM concentrations of the library, 4-*t*-butylbenzyl azide (1 mM) and Cu(MeCN)_4_PF_6_-TBTA (0.4 mM) in dichloromethane at room temperature for 48 h. UPLC conditions: C4 column at 40 °C using a 30–100% gradient of THF/formic acid (0.1%) in water/formic acid (0.1%) over 4 min, then 100% THF/formic acid (0.1%) over 2 min. (c) Schematic representation of the structures of the macrocyclic **D6A6** duplexes.

EIC screening was carried out by searching the total ion chromatogram across all retention times for signals with *m*/*z* values that matched the value calculated for the [M + 5H]^5+^ ion of the relevant macrocyclic duplex to within 1 Da. Only ions with an odd charge can be used to unambiguously identify macrocyclic duplexes because ions with an even charge may have the same *m*/*z* value as one of the macrocyclic single stranded products. The validity of this methodology was tested using the **zDDDDDDy**•zD*AAAAAAy experiment shown in Figure 5b, where the macrocyclic duplex that was unambiguously identified in the UPLC trace was the only hit observed in the EIC screen (see Figures S29 and S30). Figure 6 shows examples of EIC screening of the mixed-sequence libraries for potential macrocyclic duplex products with different compositions of phenol and phosphine oxide recognition units (see Figure S31 for EIC traces of all possible compositions). The only hit was the macrocyclic duplex composed of six phenols and six phosphine oxides (**D6A6**) in library **zDXXXXAy**. The ESI-MS recorded at the retention time identified in the **D6A6** EIC (3 min) shows a series of peaks that correspond to the 3+, 4+, 5+, 6+ and 7+ ions of the **D6A6** macrocyclic duplex (see Figure S32). Figure 6a shows that no signal was observed in library **zDXXXXAy** for the single base mismatch compositions (**D5A7** and **D7A5**), and Figure 6b shows that macrocyclic duplex products were not observed in library **zDXXXXDy**. Although oligomers with an appropriate composition to form **D6A6** products do exist in library **zDXXXXDy**, none of these oligomers have complementary sequences, and the **D6A6** product was not observed.

These results provide good evidence that the formation of macrocyclic **D6A6** duplexes in library **zDXXXXAy** is a consequence of selective high-fidelity duplex formation between fully complementary sequences that make six intermolecular H-bonds. Note that none of the homo-oligomers were present in this library, so the **D6A6** duplexes are due to sequence-selective duplex formation between two mixed sequence oligomers. The UPLC traces indicate that in addition to the macrocyclic single stranded products and macrocyclic duplexes, other species are present in the product mixtures obtained in the trapping experiments (see Figure S28). We assume that higher order oligomers are formed, but we have not been able to assign structures based on ESI-MS. Although identification of the **D6A6** macrocyclic duplex confirms that sequence-selective duplex formation takes place in these mixtures, we cannot rule out the presence of different types of supramolecular assembly, where the azide and alkyne groups are not close in space, resulting in higher order oligomeric products.

With an efficient method for synthesis of specific sequences and demonstration of sequence-selective duplex formation, these experiments establish the REMO architecture as a promising platform for the development of synthetic polymers where structure and function can be programmed using sequence.^33^

## Conclusions

REMO are synthetic polymers that feature an alternating 1,3,5-triazine-piperazine backbone and two different side-chains equipped with either a phenol or phosphine oxide recognition unit. The side-chains encode sequence information and carry H-bonding sites that confer function. An automated method for SPS of REMO of any specified sequence has been developed starting from dichlorotriazine monomer building blocks. Complementary homo-oligomers **zDDDDDDy** and zD*AAAAAAy were synthesized and shown to form a stable duplex in nonpolar solvents by NMR denaturation experiments. The zD*AAAAAAy•**zDDDDDDy** duplex was covalently trapped by equipping the ends of the oligomers with an azide and an alkyne group and using a CuAAC reaction. The SPS methodology was used to synthesize mixed sequence libraries by using a mixture of two different dichlorotriazine building blocks in each coupling cycle of oligomer synthesis. The resulting libraries contained statistical mixtures of all possible sequences. The self-assembly properties of these libraries were screened by using the CuAAC reaction to trap any duplexes present. In mixed sequence libraries of 6-mers, the trapping experiments suggest that only sequence-complementary oligomers formed duplexes at micromolar concentrations in dichloromethane. Synthetic polymers where function is encoded as a linear sequence of building blocks provide a new design space for chemistry. One of the most important functions found in nature is nucleic acid replication, and the sequence-selective duplex assembly described here suggests that the REMO architecture has a similar potential.

## Supporting Information Available

Materials and methods, detailed synthetic procedures, characterization including NMR spectra and HRMS of all compounds, NMR titration and denaturation data, UPLC, mass spectra, and EIC traces for trapping experiments (PDF)
