# Supplementary material for: Selective Duplex Formation in Mixed Sequence Libraries of Synthetic Polymers
Source: J Am Chem Soc. 2024 Mar 26;146(13):9326–34. doi: 10.1021/jacs.4c01381 (PMC10995991; doi:10.1021/jacs.4c01381)
Supplement: Supplementary file 1 — ja4c01381_si_001.pdf [file ja4c01381_si_001.pdf]

Selective Duplex Formation in Mixed Sequence Libraries of Synthetic Polymers

Mohit Dhiman,[1] Ronan Cons,[1] Oliver N. Evans,[1] Joseph T. Smith,[1] Cecilia J. Anderson,[1] Rafel Cabot,[1] Daniil O. Soloviev,[1] Christopher A. Hunter [1,*]

[1]Yusuf Hamied Department of Chemistry, University of Cambridge, Cambridge CB2 1EW, U.K. Email: herchelsmith.orgchem@ch.cam.ac.uk

Supplementary Information

1. General Experimental Details S2
2. Synthesis of Building Blocks S3
3. Protocols for Automated SPS S13
4. Oligomer Characterisation S15
5. Characterisation of Oligomer Libraries S23
6. NMR Experiments S27
7. Duplex Trapping Experiments S34

1. General Experimental Details

All reagents and materials used in the syntheses described were bought from commercial sources and used without prior purification. Dry solvents were obtained from a Grubbs PS-MD-5 solvent purification system and used with no further degassing. Thin layer chromatography (TLC) was carried out using silica gel 60F (Merck) on glass plates. LCMS analyses of samples were performed using a Waters Acquity H-class UPLC coupled with a single quadrupole Waters SQD2. Two different UPLC columns were used: an Acquity UPLC CSH C18 Column (130 Å, 1.7 µm, 2.1 mm x 50 mm), and an Acquity UPLC PRM PR BEH C4 Column (300 Å, 1.7 µm, 2.1 mm x 50 mm).

Purification of compounds by silica column chromatography were performed using an automated system (Combiflash® Rf+ or Combiflash® Rf+ Lumen) with pre-packaged silica cartridges (25 µm or 50 µm PuriFlash® columns). All NMR spectra were recorded using a Bruker 500 MHz Avance III Smart Probe Spectrometer, a Bruker 400 MHz Avance III HD Spectrometer, a Bruker 400 MHz Avance III HD Smart Probe Spectrometer, or a Bruker 400 MHz Neo Prodigy Spectrometer at 298 ± 0.1 K. The residual ^{1}H form of the solvent was used as the internal standard for referencing. In $CDCl_3$, the ^{1}H spectra were referenced to δ 7.26 ppm and ^{13}C spectra referenced to δ 77.16 ppm. In DMSO-d_6, the ^{1}H spectra were referenced to δ 2.50 ppm and ^{13}C spectra referenced to δ 39.52 ppm. Chemical shifts (δ) are quoted in ppm and coupling constants (*J*) quoted in Hz. Splitting patterns are reported as: s (singlet), bs (broad singlet), d (doublet), t (triplet), q (quartet) and m (multiplet). FT-IR spectra were collected with an ALPHA FT-IR Spectrometer from Bruker. HRMS spectra were recorded using a Waters SQD2 with Waters H-Class UPLC, equipped with a Waters Acquity UPLC BEH C18 Column (130 Å, 1.7µm, 2.1 mm x 50 mm).

UV-vis spectra were collected on an Agilent Cary 60 UV-vis spectrophotometer controlled by Cary WinUV software.

2. Synthesis of Building Blocks

Synthesis of Compounds 1 and 2

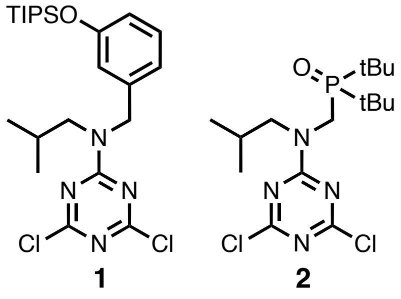

Compounds **1** and **2** were synthesised according to the literature procedure found at:

Troselj, P., Bolgar, P., Ballester, P. & Hunter, C. A. High-Fidelity Sequence-Selective Duplex Formation by Recognition-Encoded Melamine Oligomers. *J. Am. Chem. Soc.* **143**, 8669–8678 (2021).

Synthesis of Compound 3

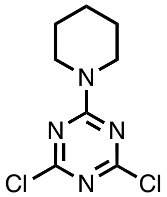

3

To a solution of cyanuric chloride (2.30 g, 12.5 mmol) in THF (90 mL) at -78 °C was added dropwise a solution of piperidine (1.0 mL, 0.85 g, 10.0 mmol) and DIPEA (3.5 mL, 2.59 g, 20.0 mmol) in THF (20 mL). The solution was stirred at -78 °C for 2h. The solvent was removed *in vacuo* and the residue was dissolved in EtOAc. The solution was washed with 0.1 M HCl (3x) and brine before the organic phase was dried over $MgSO_4$, then the solvent was removed *in vacuo* to yield the crude product. The crude was purified by flash chromatography (SiO_2, 0-30% gradient of EtOAc in 40-60 petroleum ether) to yield the pure product **3** as a white solid (2.29 g, 9.87 mmol, 80%).

^{1}H NMR (400 MHz, chloroform-d): δ_H 3.85-3.78 (m, 2H), 1.76-1.68 (m, 1H), 1.68-1.60 (m, 2H);
HRMS (ES+): calculated for $C_8H_{11}Cl_2N_4$ $[M+H]^+$ 233.0361, found 233.0358 $[M+H]^+$.

The spectroscopic data matches that previously reported in the literature at:

Almalioti, F. *et al.* Convenient syntheses of cyanuric chloride-derived NHC ligands, their Ag(I) and Au(I) complexes and antimicrobial activity. *Dalton Trans.* **42**, 12370–12380 (2013).

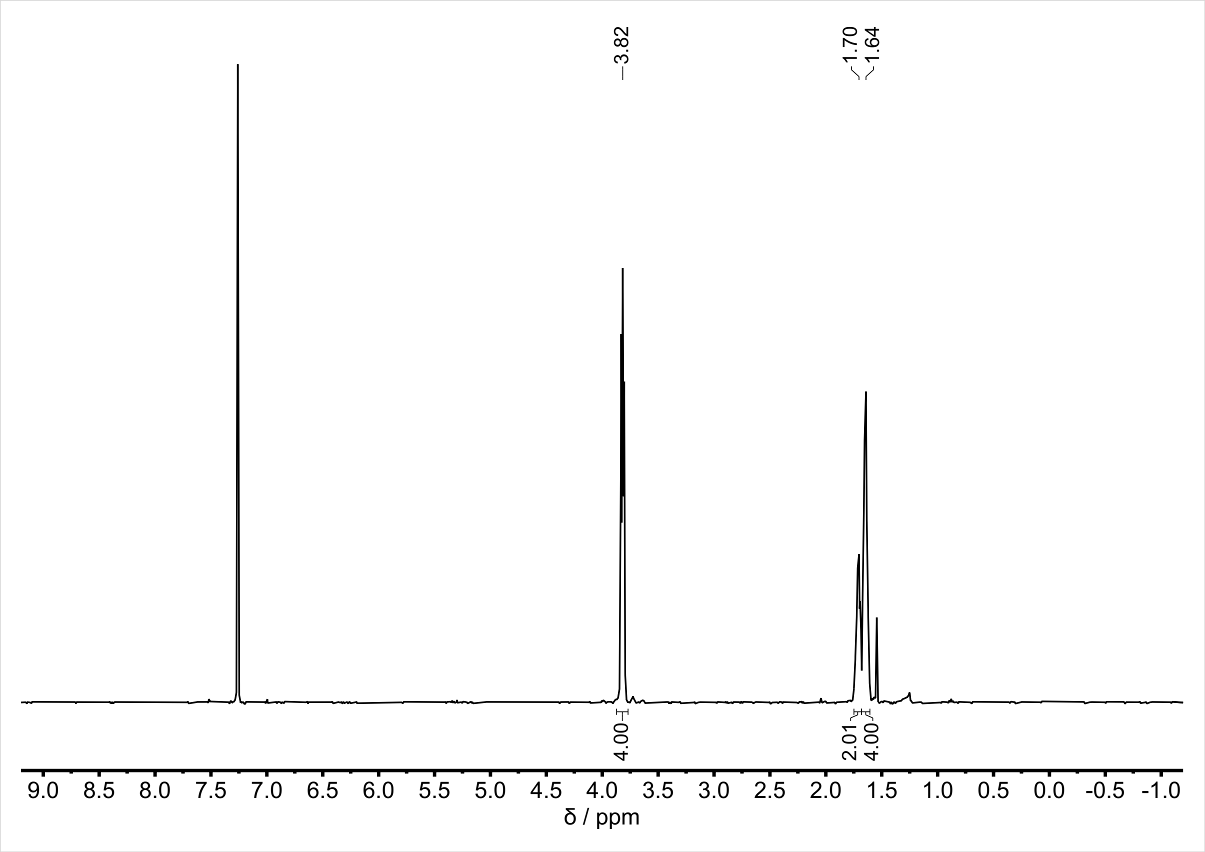

Figure S1: ^{1}H NMR (400 MHz, chloroform-d) spectrum of **3**.

Synthesis of Compound 4

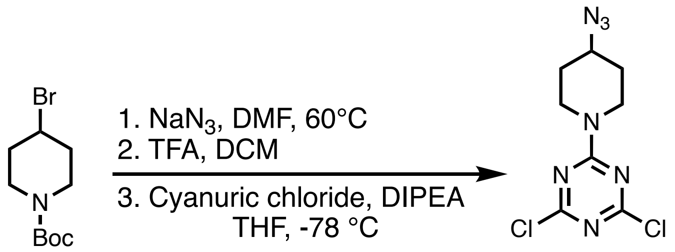

4

A solution of 1-Boc-4-bromopiperidine (400 mg, 1.51 mmol) and sodium azide (197 mg, 3.03 mmol) in DMF (5 mL) was stirred overnight at 60 °C under N_2 atmosphere. The mixture was diluted with water (20 mL) then extracted with EtOAc (3x). The combined organic layers were washed with water (3x), 5% LiCl soln. (3x) and brine. The organic phase was dried over $MgSO_4$ and then the solvent was removed *in vacuo* to obtain a colourless oil.

The obtained product was dissolved in DCM (6 mL) before TFA (2 mL) was added and the mixture was stirred at r.t. for 30 mins. The solvent and reagent were removed by flushing the mixture under N_2 to obtain a colourless oil.

To a solution of cyanuric chloride (419 mg, 2.27 mmol) in THF (40 mL) at -78 °C was added dropwise a solution of the crude obtained in the previous step and DIPEA (1.05 mL, 781 mg, 6.04 mmol) in THF (10 mL). The solution was stirred at -78 °C for 1h. The solvent was removed *in vacuo* and the residue was dissolved in EtOAc. The solution was washed with 0.1 M HCl (3x) and brine before the organic phase was dried over $MgSO_4$, then the solvent was removed *in vacuo* to yield the crude product. The crude was purified by flash chromatography (SiO_2, 0-20% gradient of EtOAc in 40-60 petroleum ether) to yield the pure product **4** as a white solid (316 mg, 1.15 mmol, 76% over three steps).

^{1}H NMR (400 MHz, chloroform-d): δ_H 4.19-4.09 (m, 2H), 3.84-3.73 (m, 1H), 3.73-3.62 (m, 2H), 2.02-1.90 (m, 2H), 1.76-1.63 (m, 2H);
^{13}C NMR (101 MHz, chloroform-d): δ_C 170.5, 164.0, 56.7, 41.5, 30.4;
HRMS (ES+): calculated for $C_8H_9Cl_2$ 274.0369 $[M+H]^+$, found 274.0371 $[M+H]^+$;
FT-IR (ATR): ν_{max} /cm^{-1} 2931, 2874, 2092, 1572, 1475, 1349, 1326, 1232, 1194, 1170, 1154, 1134, 1093, 1066.

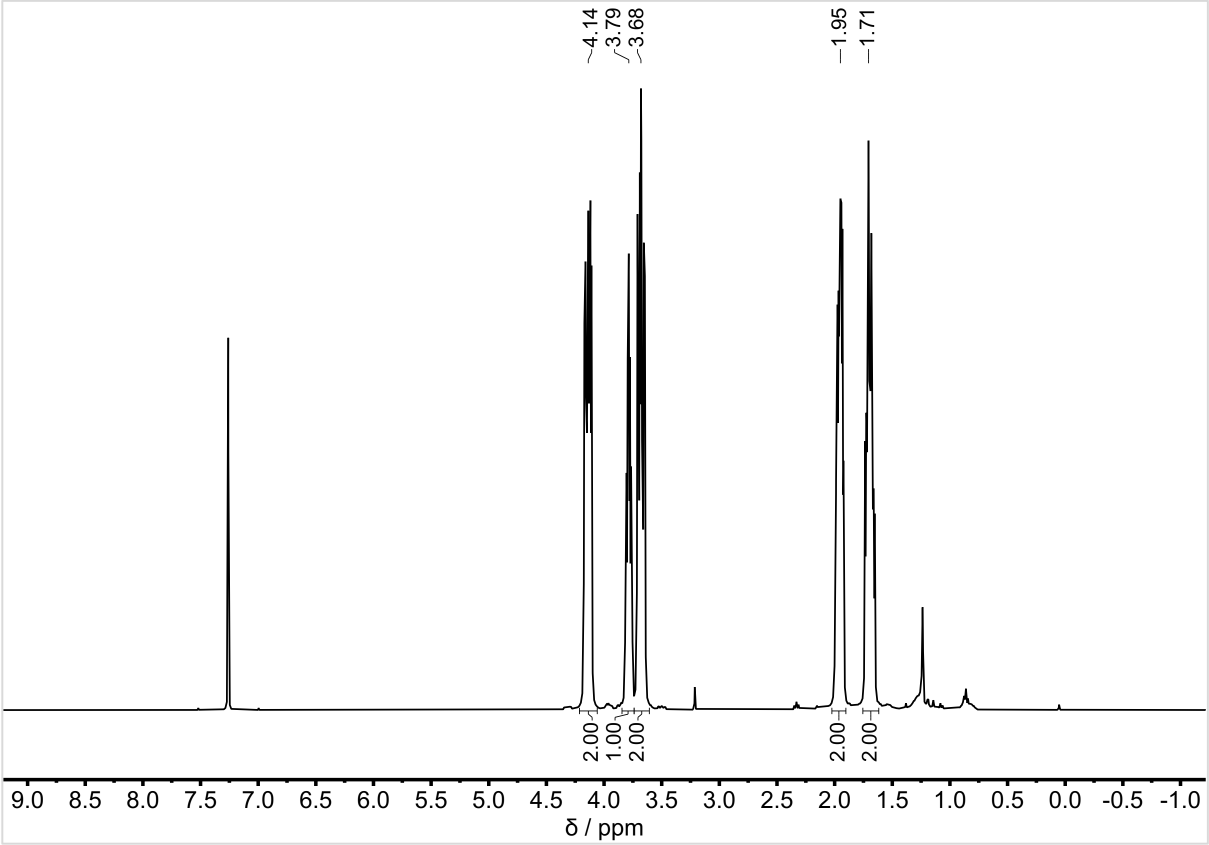

Figure S2: ^{1}H NMR (400 MHz, chloroform-d) spectrum of **4**.

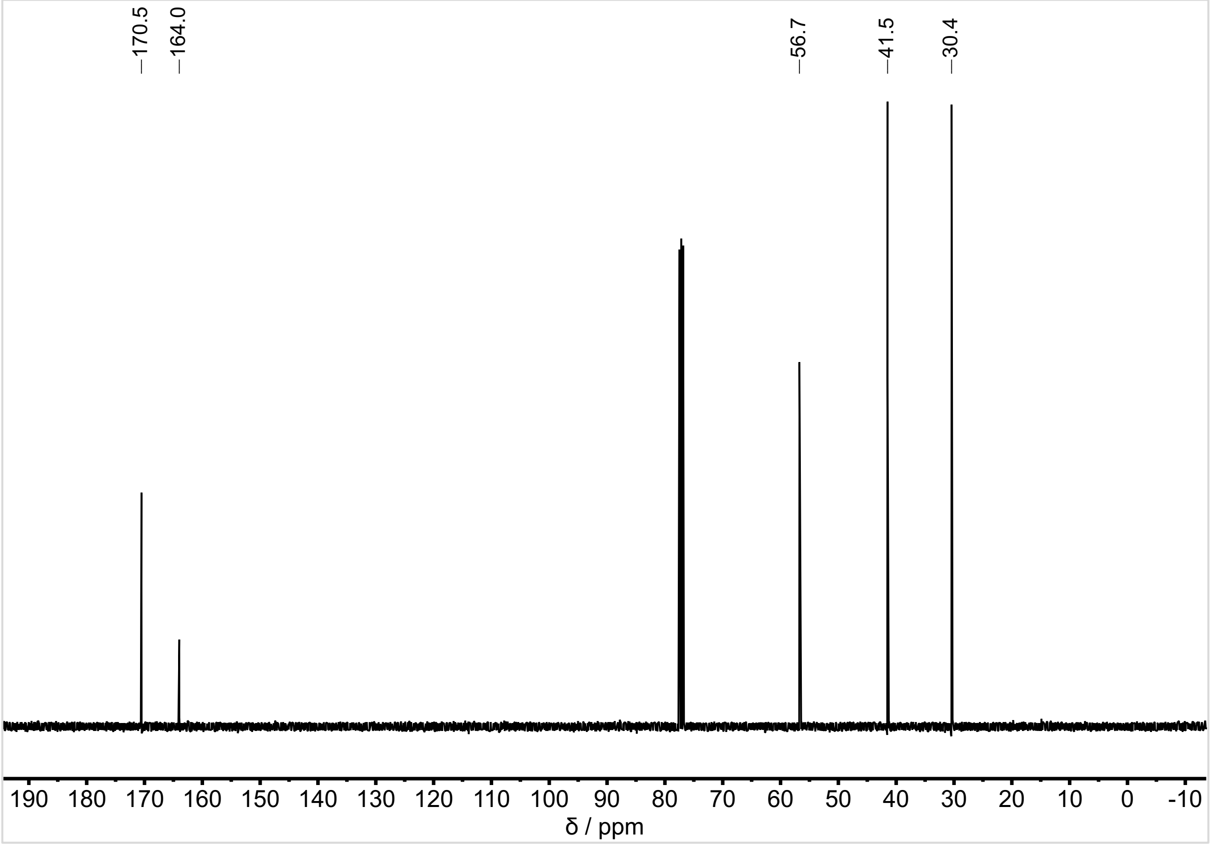

Figure S3: ^{13}C NMR (101 MHz, chloroform-d) spectrum of **4**.

Synthesis of Compound 5

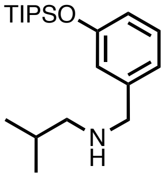

Compound **5** was synthesised according to the literature procedure found at:

Troselj, P., Bolgar, P., Ballester, P. & Hunter, C. A. High-Fidelity Sequence-Selective Duplex Formation by Recognition-Encoded Melamine Oligomers. *J. Am. Chem. Soc.* **143**, 8669–8678 (2021).

Synthesis of Compound 6

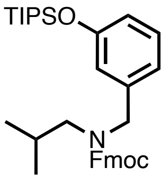

To a solution of **5** (0.720 g, 2.15 mmol) in DCM (8 mL) at 0 °C was added Fmoc chloride (0.610 g, 2.36 mmol), then triethylamine (0.239 g, 0.33 mL, 2.36 mmol) was added dropwise. The reaction was stirred for 2 h at r.t. before the mixture was washed with 1 M K_2CO_3 solution and brine. The organic phase was dried over $MgSO_4$ and then the solvent was removed *in vacuo* to yield the crude product as a yellow oil. The crude was purified by flash chromatography (SiO_2, 0-20% gradient of EtOAc in 40-60 petroleum ether) to yield the product **6** (1.16 g, 2.08 mmol, 97%) as a colourless oil.

The NMR spectra are consistent with the presence of two slowly exchanging rotamers in solution. Where rotamers are distinguishable, the corresponding peaks are listed together.

^{1}H NMR (400 MHz, chloroform-d): δ_H 7.75 & 7.73 (d, J = 7.5 Hz, 2H, rotamers), 7.60 & 7.46 (d, J = 7.2 Hz, 2H, rotamers)7.42-7.33 (m, 2H), 7.32 & 7.23 (t, J = 7.2 Hz, 1H), 7.17-7.12 (m, 1H), 6.81-6.75 (m, 1H), 6.75–6.67 (m, 2H), 4.57 & 4.44 (d, J = 5.5 Hz, 2H, rotamers), 4.45 & 4.43 (s, 2H), 4.25 & 4.20, (t, J = 5.5 Hz, 1H), 3.11 & 2.79 (d, J = 7.4 Hz, 2H, rotamers), 1.98 & 1.69 (non, J = 6.7 Hz, 1H), 1.23 (m, 3H), 1.07 (d, J = 7.2 Hz, 18H), 0.89 & 0.69 (d, J = 6.7 Hz, 6H);

^{13}C NMR (101 MHz, chloroform-d): δ_C 157.1 & 156.7 (rotamers), 156.5 & 156.3 (rotamers), 144.3 & 144.2 (rotamers), 141.6 & 141.4 (rotamers), 139.4 & 139.4 (rotamers), 129.7 & 129.6 (rotamers) 127.7 & 127.2 (rotamers), 125.2 & 124.9 (rotamers), 120.5 & 119.7 (rotamers), 120.0, 119.4 & 118.6 (rotamers), 118.9 & 118.8 (rotamers), 67.6 & 67.1 (rotamers), 54.4 & 53.3 (rotamers), 50.8 & 50.4 (rotamers), 47.6 & 47.5 (rotamers), 27.1, 20.2 & 20.0 (rotamers), 18.0, 12.8;

HRMS (ES+): calculated for $C_{35}H_{47}NO_3Si$ 557.3320 $[M+H]^+$, found 557.3272 $[M+H]^+$;

FT-IR (ATR): ν_{max} /cm^{-1} 2947, 2868, 1694, 1603, 1586, 1486, 1464, 1448, 1421, 1264, 1243, 1149, 1093, 1003, 981, 883, 822, 736.

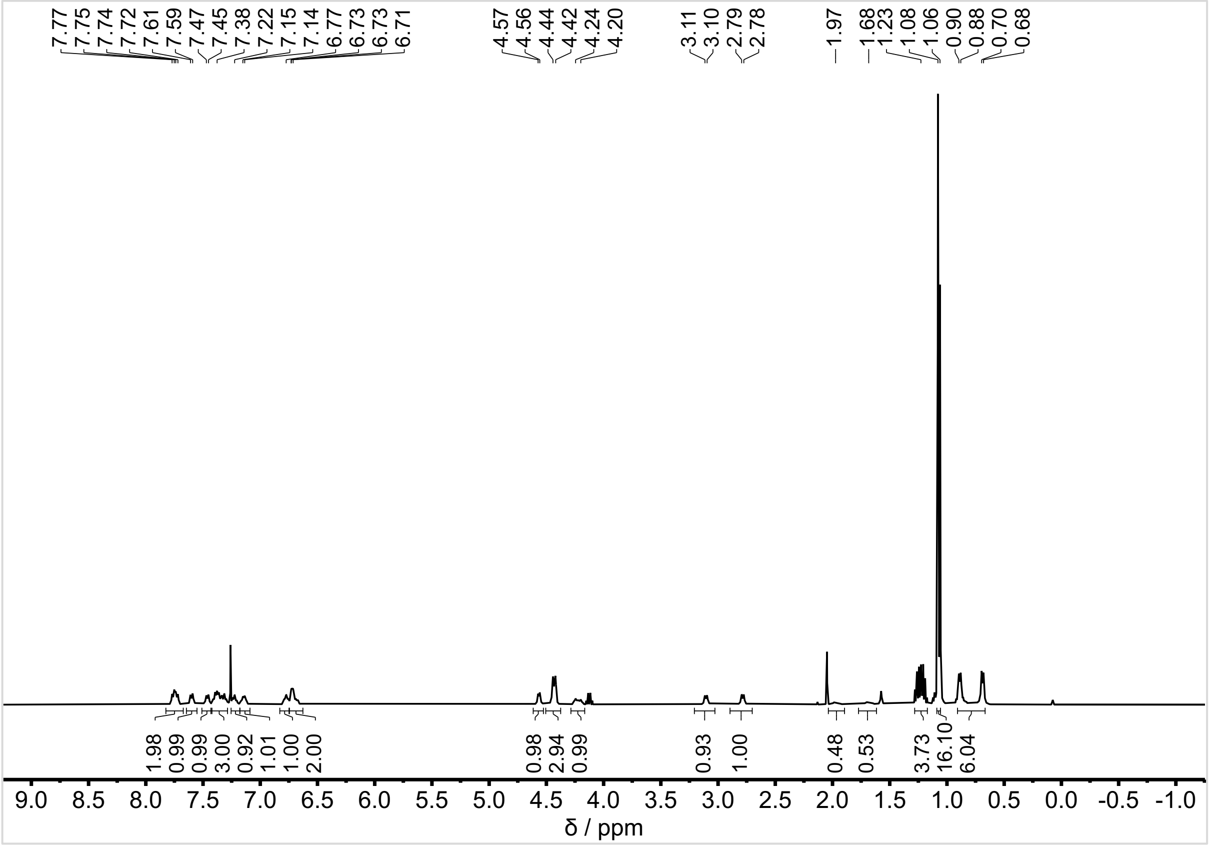

Figure S4: ^{1}H NMR (400 MHz, chloroform-d) spectrum of **6**.

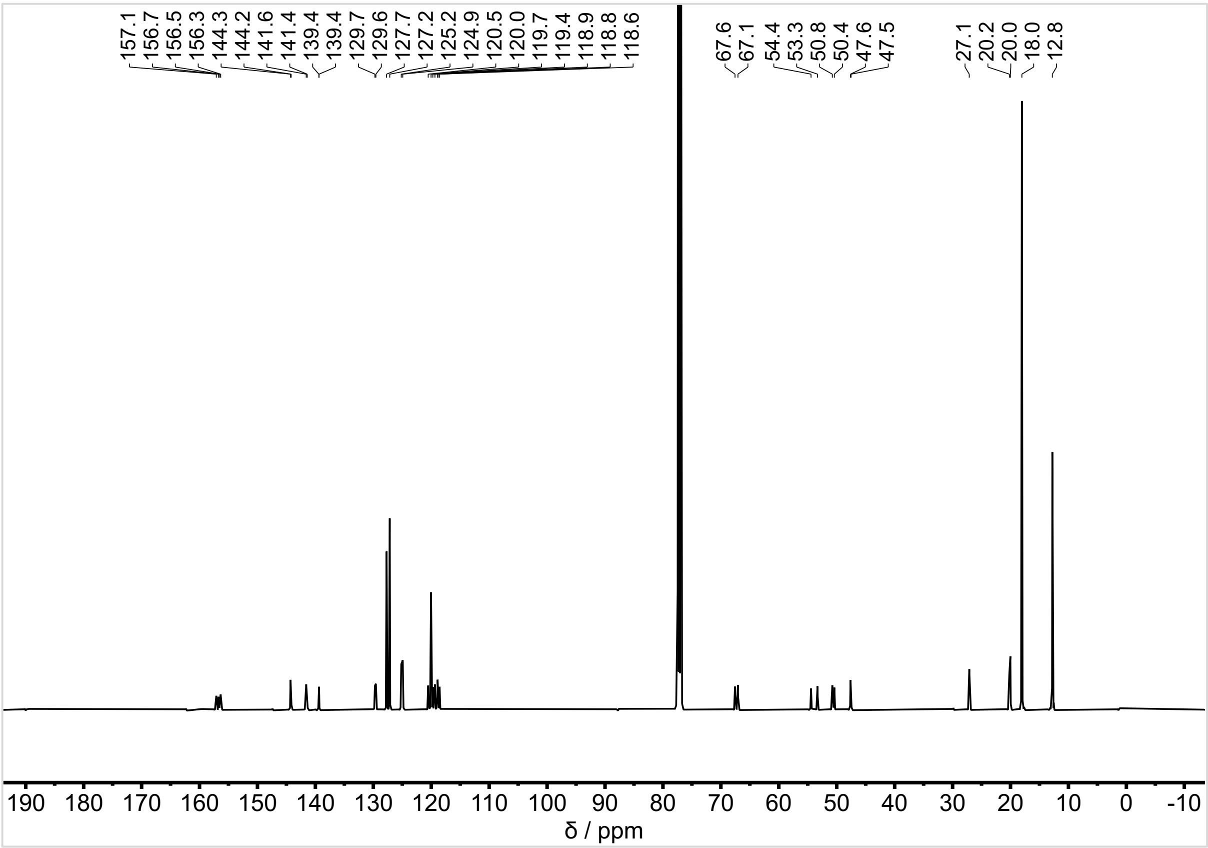

Figure S5: ^{13}C NMR (101 MHz, chloroform-d) spectrum of **6**.

Synthesis of Compound 7

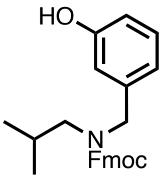

6 (1.00g, 1.79 mmol) was dissolved in THF (15 mL) and the solution was acidified to pH 3 by adding 1M AcOH. TBAF (3.6 mL, 3.59 mmol, 1M in THF) was added dropwise and the reaction mixture was stirred at r.t. until complete conversion of the starting material, as monitored by LCMS (approx. 2 h). After completion, the reaction was quenched with 5% aq. soln. HCl and extracted with EtOAc (3x) followed by washing with 5% aq. soln. HCl (3x) and brine. The organic phase was dried over $MgSO_4$ and the solvent was removed *in vacuo* to yield the crude product. The crude was purified by flash chromatography (SiO_2, 0-70% gradient of EtOAc in 40-60 petroleum ether) to yield the pure product **7** (0.592 g, 1.47 mmol, 82%) as a colourless foam.

The NMR spectra are consistent with the presence of two slowly exchanging rotamers in solution. Where rotamers are distinguishable, the corresponding peaks are listed together.

^{1}H NMR (400 MHz, chloroform-d): δ_H 7.75 & 7.70 (d, J = 7.5 Hz, 2H, rotamers), 7.54 & 7.40 (d, J = 7.5 Hz, 2H, rotamers), 7.39-7.32 (m, 2H), 7.29 & 7.21 (t, J = 7.5 Hz, 2H, rotamers), 7.18-7.10 (m, 1H), 6.80-6.72 (m, 1H), 6.68-6.59 (m, 1H), 6.69 & 6.52 (s, 1H, rotamers), 6.43 (br s, 1H), 4.57 & 4.47 (d, J = 5.5, J = 6.2 Hz, 2H, rotamers), 4.38 & 4.33 (s, 2H, rotamers), 4.21-4.14, (m, 1H), 3.08 & 2.73 (d, J = 7.5 Hz, 2H, rotamers), 1.95 & 1.63 (non, J = 6.6 Hz, 1H), 0.84 & 0.65 (d, J = 6.6 Hz, 6H);

^{13}C NMR (101 MHz, chloroform-d): δ_C 157.4 & 157.0 (rotamers), 156.8 & 156.6 (rotamers), 144.1 & 144.0 (rotamers), 141.6 & 141.4 (rotamers), 139.3, 129.9 & 129.7 (rotamers) 127.7 & 127.2 (rotamers), 125.1 & 124.8 (rotamers), 120.5 & 119.7 (rotamers), 120.0, 119.8 & 118.9 (rotamers), 114.7, 114.7 (rotamers), 114.5 & 113.5 (rotamers), 54.4 & 53.3 (rotamers), 67.5 & 67.0 (rotamers), 54.6 & 53.5 (rotamers), 50.9 & 50.3 (rotamers), 47.5 & 47.4 (rotamers), 27.1, 20.1 & 20.0 (rotamers);

HRMS (ES+): calculated for $C_{26}H_{27}NO_3$ 402.2069 $[M+H]^+$, found 402.2057 $[M+H]^+$;

FT-IR (ATR): ν_{max} /cm^{-1} 3333 (br), 2958, 2926, 2871, 1671, 1601, 1590, 1478, 1451, 1426, 1388, 1366, 1249, 1149, 1094, 998, 969, 908, 879, 759, 733, 702, 648.

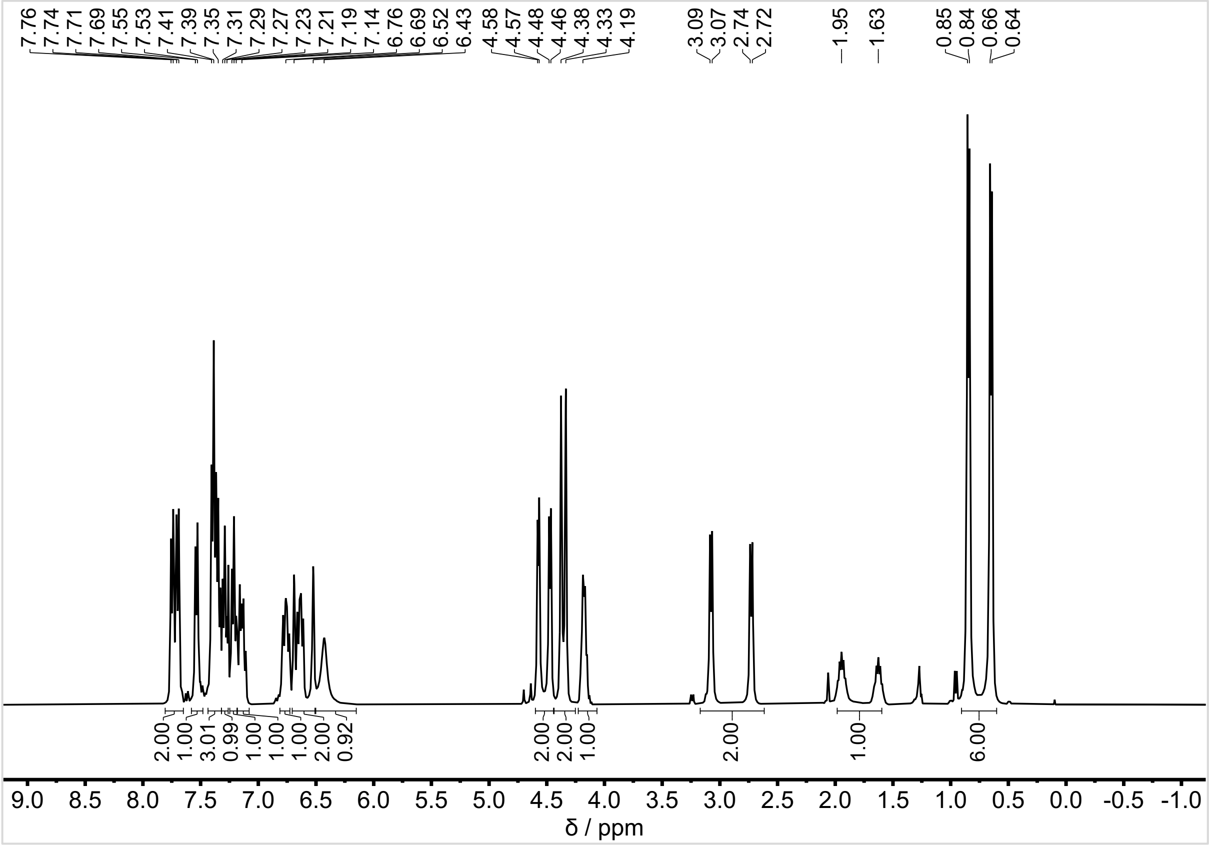

Figure S6: ^{1}H NMR (400 MHz, chloroform-d) spectrum of **7**.

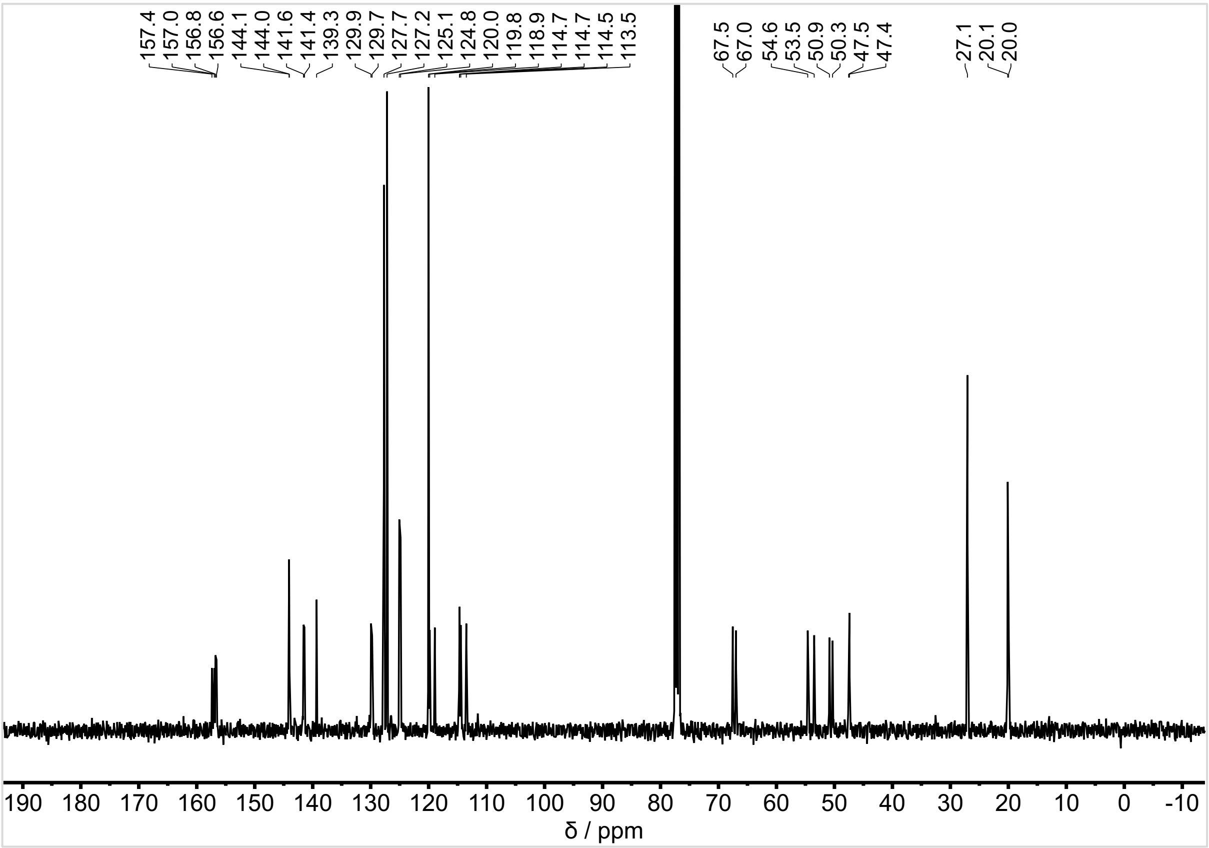

Figure S7: ^{13}C NMR (101 MHz, chloroform-d) spectrum of **7**.

3. Protocols for Automated SPS

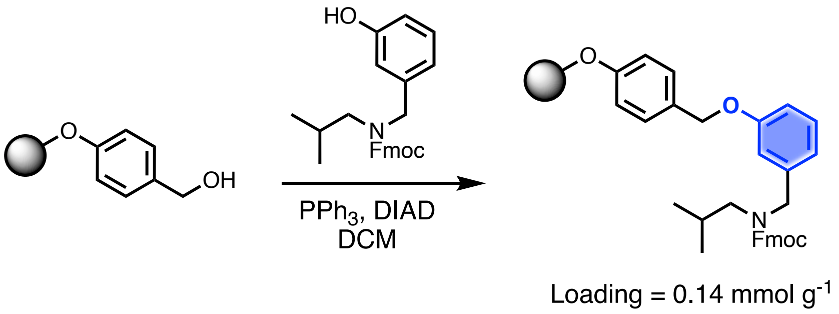

TentaGel Wang Resin (90 µm mesh) (3.00 g, 0.72 mmol based on advertised loading) was swollen in dry DCM for 30 min before a solution of **7** (0.578 g, 1.44 mmol) and triphenylphosphine (0.377g, 1.44 mmol) in dry DCM was added to the resin. A solution of diisopropyl azodicarboxylate (0.284 mL, 0.291 g, 1.44 mmol) was diluted 5-fold in dry DCM then added dropwise to the resin. The resin was agitated at r.t. overnight, and then washed alternatingly with DCM (5x) and DMF (5x) to yield the functionalised resin.

Quantification of resin loading: Functionalised Wang Resin was treated with a solution of DBU in DMF (2 mL, 2 vol. %) and agitated for 30 min. 1 mL of the solution was removed from the resin and diluted with 4 mL acetonitrile. A 1 mL aliquot of the resultant solution was taken and diluted to 12.5 mL with acetonitrile. The absorbance of the DBU-fulvene adduct (λ = 304 nm, ε = 9254 M^{-1} cm^{-1}) was measured to estimate the resin loading (0.14 mmol g^{-1}).

.

General methods using CEM Liberty Blue Automated Synthesiser: Heated automated Solid-Phase Organic Synthesis (SPS) was performed on a CEM Liberty Blue automated synthesizer on a 50 μmol scale. Solutions of piperazine (0.7 M) in DMF, dichlorotriazines **1**, **2**, **3**, **4** or a mixture for library synthesis (0.125 M) in DMF, and DIPEA (0.5 M) in DMF were prepared for coupling. General synthetic protocols performed were:

Fmoc deprotection: The loaded Wang resin was agitated in a solution of piperazine in DMF (7 mL, 0.7 M, 2 x 10 min). The deprotection solution was then drained and the resin was washed with DMF (4 x 5 mL).

Coupling cycle: The resin-bound oligomer was first agitated in a solution of dichlorotriazine (0.1 M, 10 eq.) and DIPEA (0.1 M, 10 eq.) in DMF (5 mL) for 10-15 mins at 90 °C. The 1st coupling solution was drained, and the resin was washed with DMF (4 x 5 mL). The resin-bound oligomer was then agitated in a solution of piperazine (5 mL, 0.7 M) in DMF for 10 or 15 min at 90 °C. The 2nd coupling solution was drained, and the resin was washed with DMF (4 x 5 mL).

Terminal coupling (manual MW-assisted SPS): The resin-bound oligomer was swollen in DMF for 15 min before being agitated in a solution of piperidine or 4-ethynylpiperidine trifluoroacetate (0.1 M) and DIPEA (1 M) in DMF (5 mL) for 30 mins at 90 °C. The coupling solution was drained, and the resin washed with DMF (4 x 5 mL).

TIPS deprotection: The resin-bound oligomer was swollen in THF for 15 min before being agitated in a solution of TBAF (5 mL, 1M in THF) at r.t. for 1h. The solution was drained, and the resin washed with MeOH and THF alternatingly (4 x 5 mL) before being subjected to another deprotection cycle.

Resin Cleavage: The resin was agitated in a mixture of TFA:TIS:DCM (90:5:5 v/v/v) at r.t. for 2 h. The resin was filtered and washed with DCM (5 x 5 mL) and then subjected to another cleavage cycle. The combined filtrates from both cleavage cycles were concentrated under N_2 flow before drying *in vacuo*.

4. Oligomer Characterisation

pDDADADDDDAp

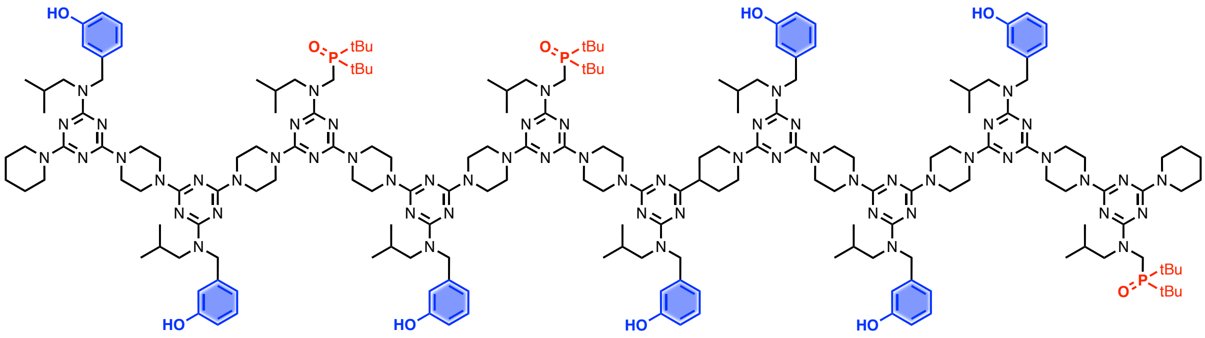

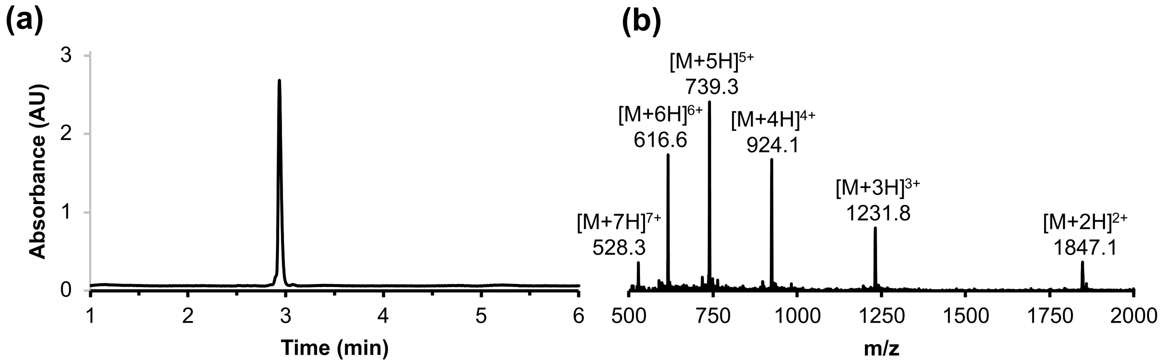

Figure S8: (a) UPLC trace of **pDDADADDDDAp**. **(b)** ESI-MS of **pDDADADDDDAp**. Calculated mass: 1847.2 $[M+2H]^{2+}$, 1231.7 $[M+3H]^{3+}$, 924.2 $[M+4H]^{4+}$, 739.3 $[M+5H]^{5+}$, 616.6 $[M+6H]^{6+}$, 528.3 $[M+7H]^{7+}$; Mass found (ESI^+): 1847.1 $[M+2H]^{2+}$, 1231.8 $[M+3H]^{3+}$, 924.1 $[M+4H]^{4+}$, 739.3 $[M+5H]^{5+}$, 616.6 $[M+6H]^{6+}$, 528.3 $[M+7H]^{7+}$. *UPLC Conditions:* C4 column at 40 °C using a 30-100% gradient of THF/formic acid (0.1%) in water/ formic acid (0.1%) over 4 minutes, then 100% THF/formic acid (0.1%) over 2 minutes.

^{31}P NMR (162 MHz, DMSO-d$_6$): δ_P 62.33;

HRMS (ES+): calculated for $C_{192}H_{291}N_{60}O_{10}P_3$ 1231.1185 $[M+3H]^{3+}$, found 1231.1201 $[M+3H]^{3+}$.

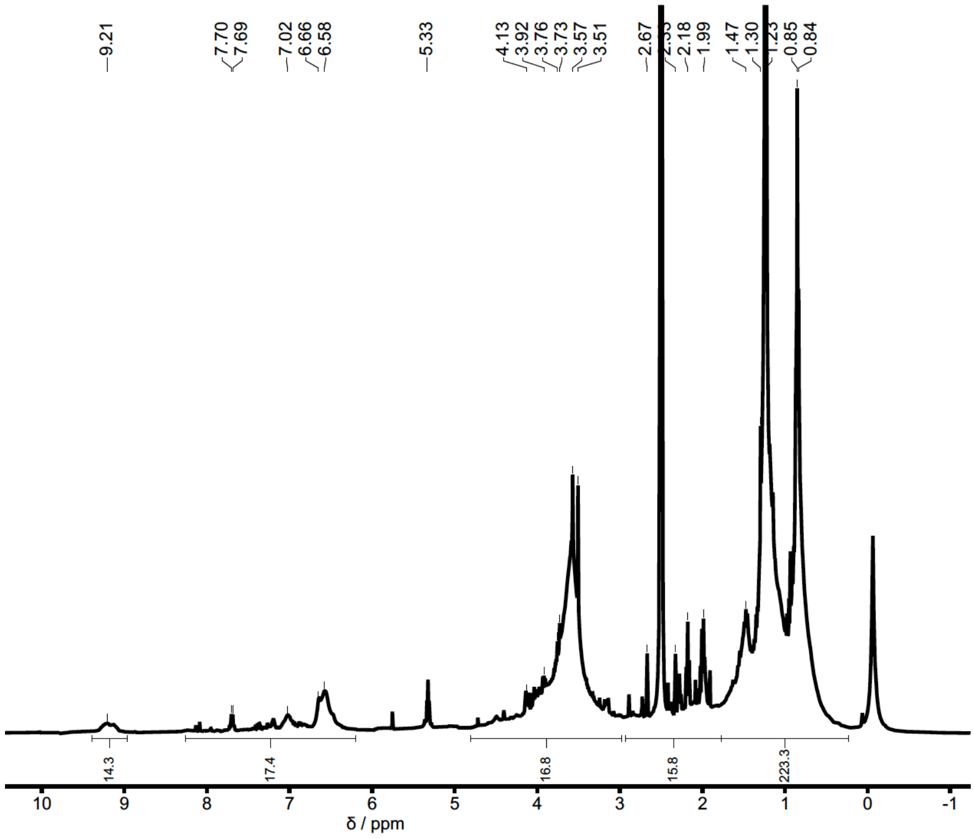

Figure S9: ^{1}H NMR (400 MHz, DMSO-d$_6$) spectrum of **pDDADADDDDAp**.

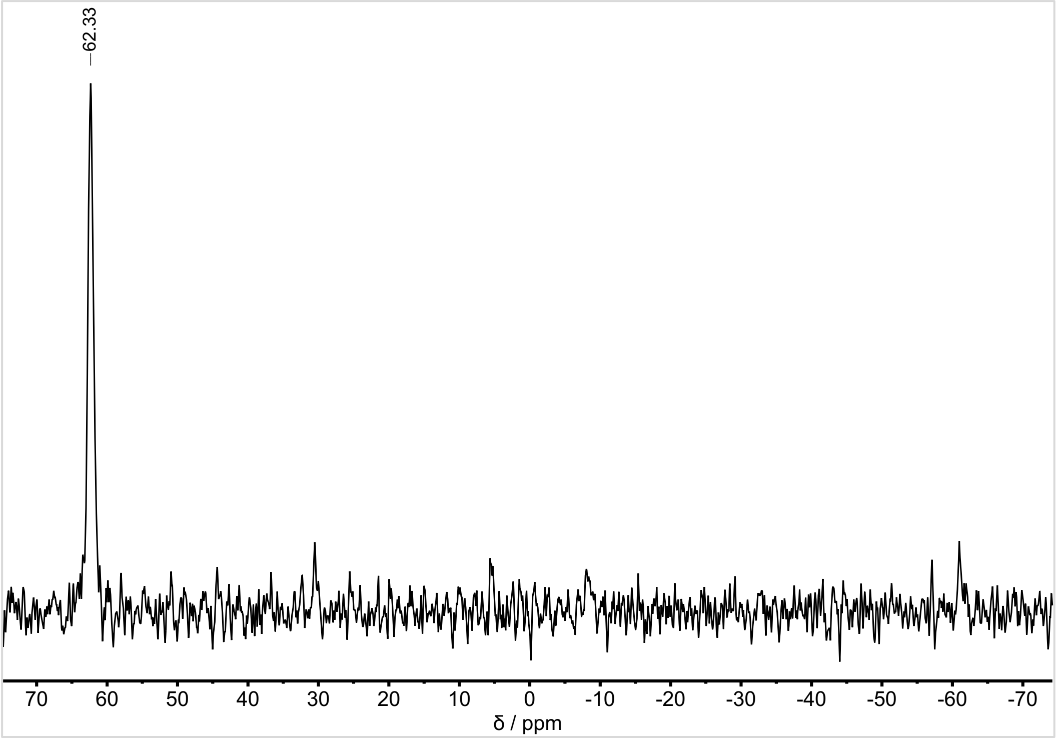

Figure S10: ^{31}P NMR (162 MHz, DMSO-d$_6$) spectrum of **pDDADADDDDAp**.

zDDDDDDy

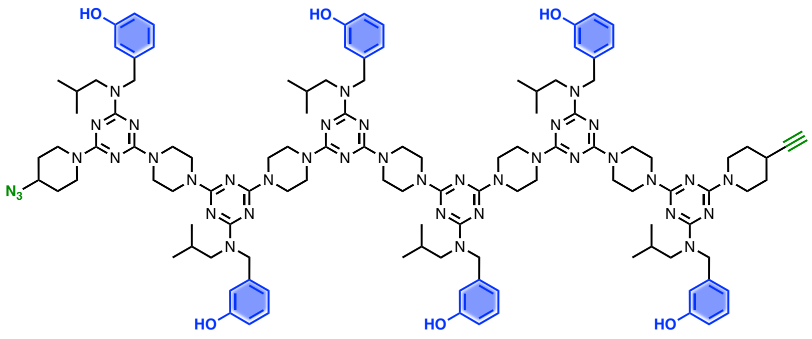

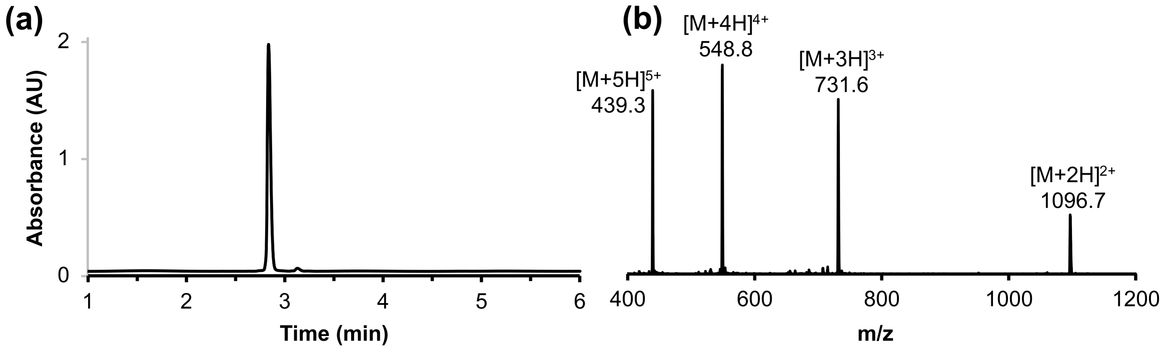

Figure S11: (a) UPLC trace of **zDDDDDDy**. **(b)** ESI-MS of **zDDDDDDy**. Calculated mass: 1096.7 $[M+2H]^{2+}$, 731.4 $[M+3H]^{3+}$, 548.8 $[M+4H]^{4+}$, 439.3 $[M+5H]^{5+}$; Mass found (ESI$^+$): 1096.7 $[M+2H]^{2+}$, 731.6 $[M+3H]^{3+}$, 548.8 $[M+4H]^{4+}$, 439.3 $[M+5H]^{5+}$. *UPLC Conditions:* C4 column at 40 °C using a 30-100% gradient of THF/formic acid (0.1%) in water/ formic acid (0.1%) over 4 minutes, then 100% THF/formic acid (0.1%) over 2 minutes.

^{1}H NMR (400 MHz, DMSO-d$_6$): δ_H 9.24 (phenol OH, 6H), 7.11-7.02 & 6.69-6.56 (aryl CH, 24H), 4.79-2.91 (NCH_2, 73H), 2.62 ((HC≡C)C<u>H</u>, 1H), 2.11-1.31 (CH_2, *i*-Bu CH & C≡CH, 15H), 0.89-0.79 (*i*-Bu CH_3, 36H);

HRMS (ES+): calculated for $C_{116}H_{155}N_{39}O_6$ 2214.2942 $[M+Na]^+$, found 2214.3022 $[M+Na]^+$.

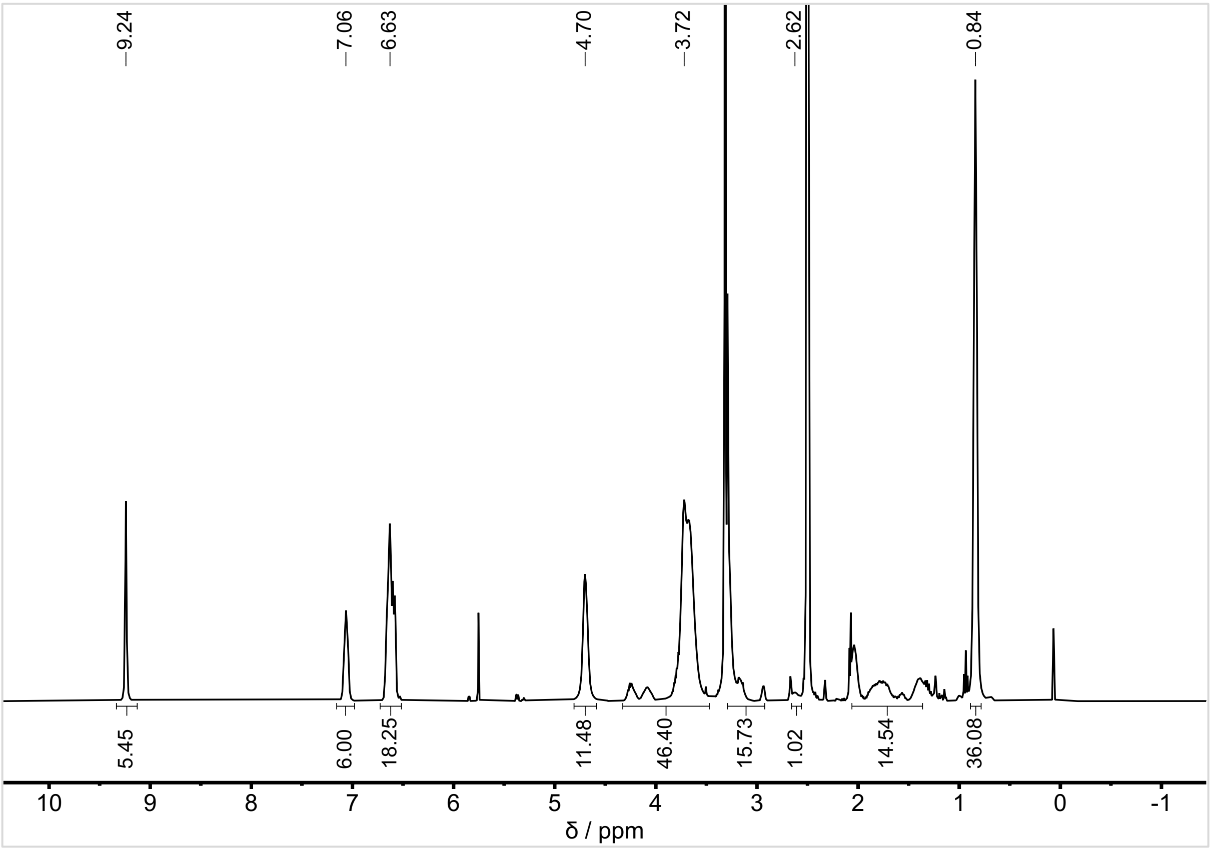

Figure S12: ^{1}H NMR (400 MHz, DMSO-d_6) spectrum of **zDDDDDDy**.

zDAAAAAAy

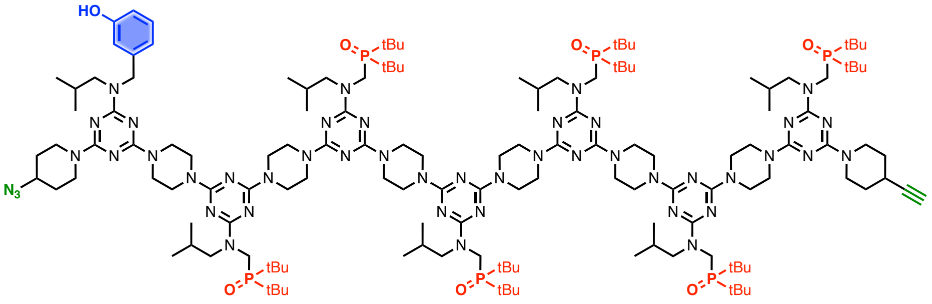

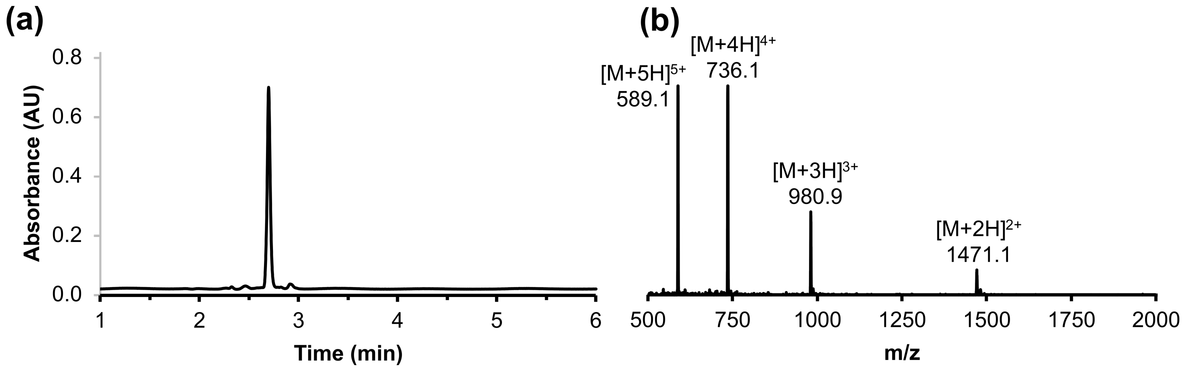

Figure S13: (a) UPLC trace of **zDAAAAAAy**. **(b)** ESI-MS of **zDAAAAAAy**. Calculated mass: 1471.0 $[M+2H]^{2+}$, 981.0 $[M+3H]^{3+}$, 736.0 $[M+4H]^{4+}$, 589.0 $[M+5H]^{5+}$; Mass found (ESI^+): 1471.1 $[M+2H]^{2+}$, 980.9 $[M+3H]^{3+}$, 736.1 $[M+4H]^{4+}$, 589.1 $[M+5H]^{5+}$. *UPLC Conditions:* C4 column at 40 °C using a 30-100% gradient of THF/formic acid (0.1%) in water/ formic acid (0.1%) over 4 minutes, then 100% THF/formic acid (0.1%) over 2 minutes.

^{1}H NMR (400 MHz, chloroform-d): δ_H 10.26 (TFA impurity), 7.17-6.63 (aryl CH, 4H) 4.82-3.11 (NCH_2, 85H), 2.78 ((HC≡C)C<u>H</u>, 1H), 2.37-1.54 (CH_2, *i*-Bu CH & C≡CH, 16H), 1.41-1.22 (*t*-Bu CH_3, 108H), 0.96-0.83 (*i*-Bu CH_3, 42H);
^{31}P NMR (162 MHz, chloroform-d): δ_P 61.77 (high chemical shift due to TFA impurity)
HRMS (ES+): calculated for $C_{146}H_{257}N_{45}O_7P_6$ 2939.9636 $[M+H]^+$, found 2939.9653 $[M+H]^+$.

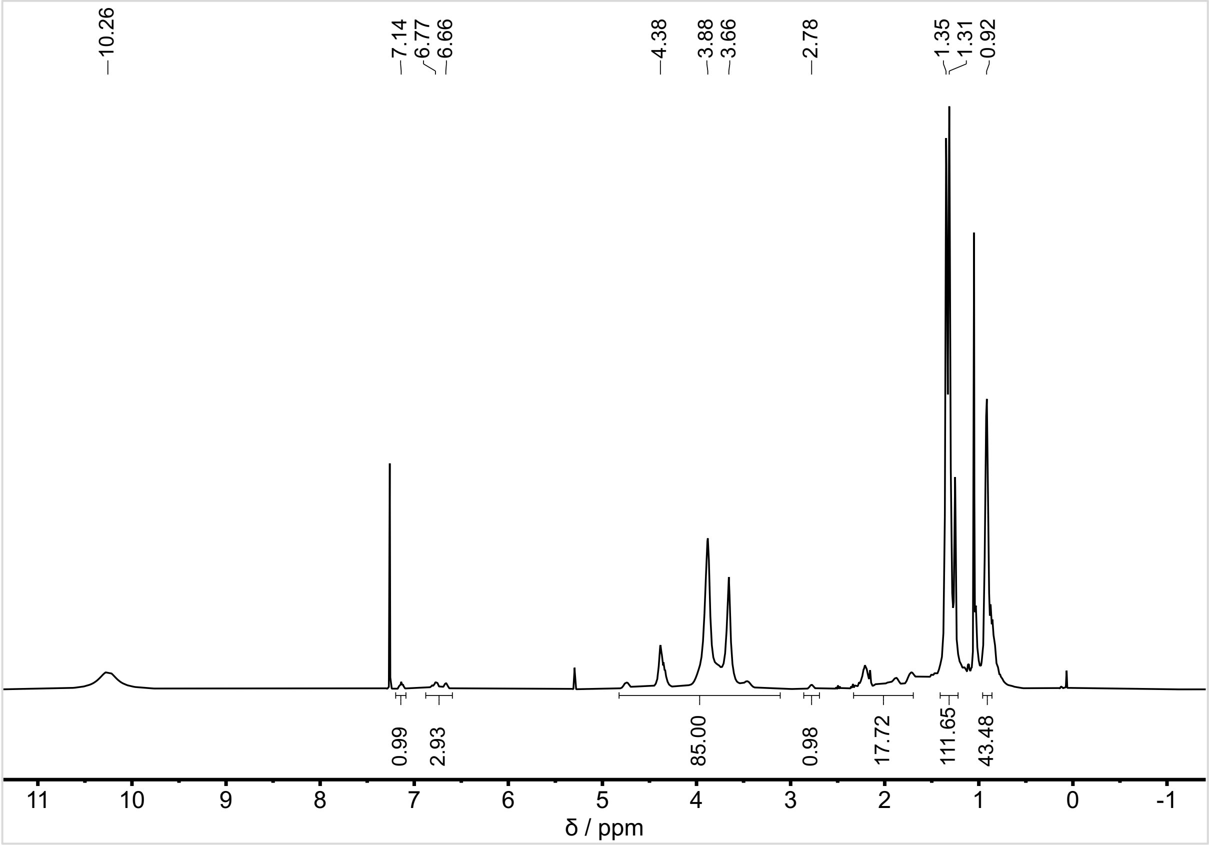

Figure S14: ^{1}H NMR (400 MHz, chloroform-d) spectrum of **zDAAAAAAy**.

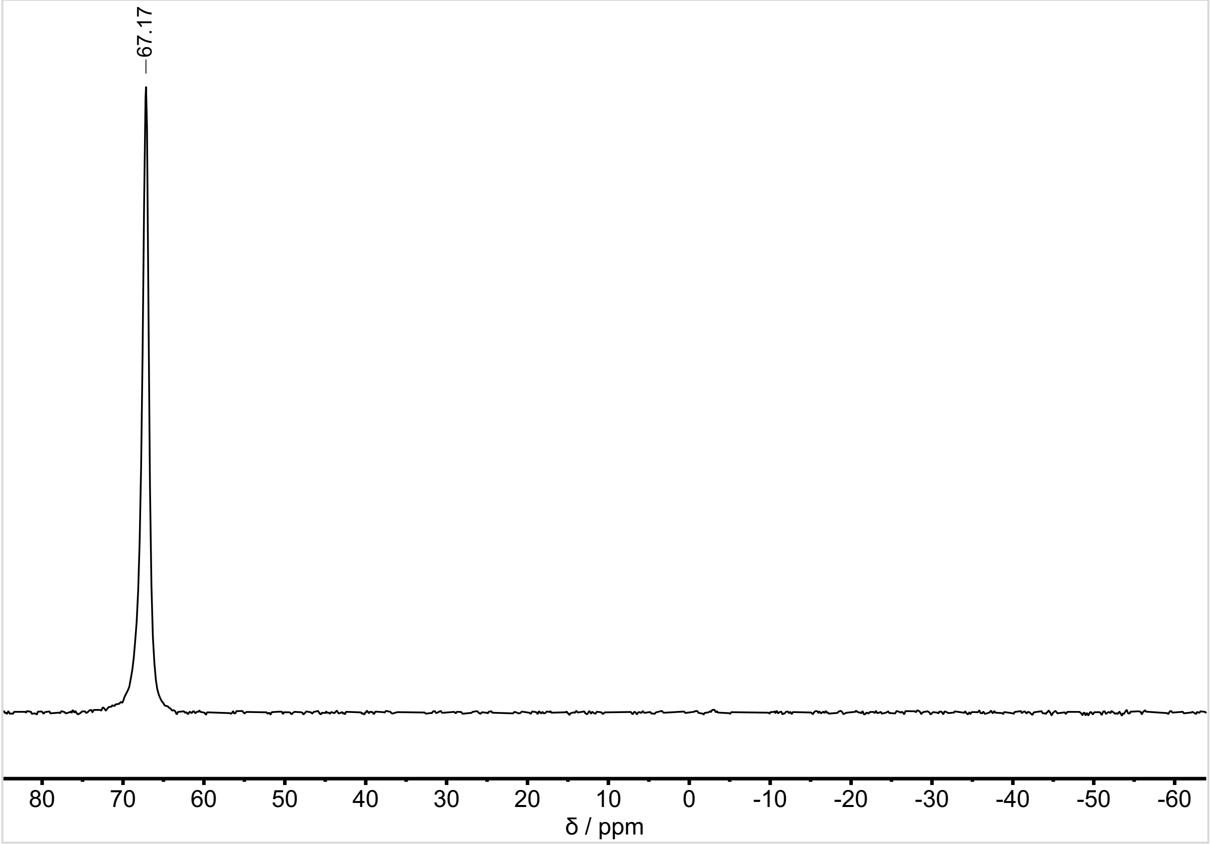

Figure S15: ^{31}P NMR (162 MHz, chloroform-d) spectrum of **zDAAAAAAy**.

zD*AAAAAAy

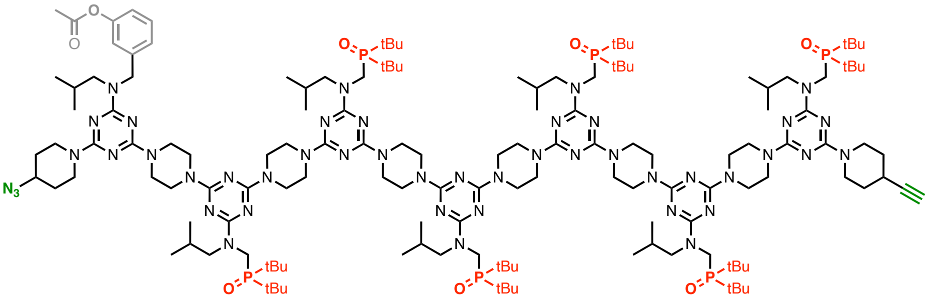

A mixture of **zDAAAAAAy** (39 mg, 0.013 mmol), glacial acetic acid (8 mg, 0.13 mmol), EDC (25 mg, 0.13 mmol) and DMAP (16 mg, 0.13 mmol) in dry THF (4 mL) was stirred at r.t. overnight. The mixture was extracted with EtOAc (3x) and washed with 1M HCl (2x) and brine. The organic phase was dried over $MgSO_4$ and the solvent was removed *in vacuo* to yield the crude product. The crude was purified by flash chromatography (SiO_2, 0-20% gradient of methanol in DCM) to yield the product **zD*AAAAAAy** (20 mg, 0.007 mmol, 51%) as a white solid.

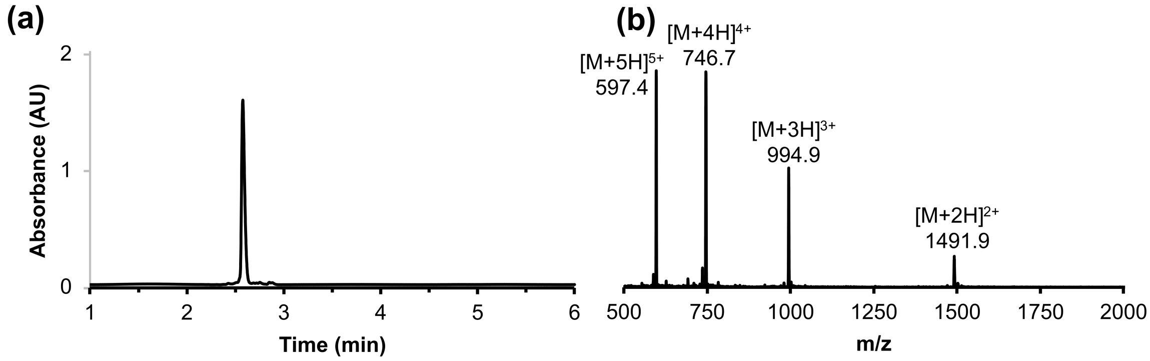

Figure S16: (a) UPLC trace of **zD*AAAAAAy**. **(b)** ESI-MS of **zD*AAAAAAy**. Calculated mass: 1492.0 $[M+2H]^{2+}$, 995.0 $[M+3H]^{3+}$, 746.5 $[M+4H]^{4+}$, 597.4 $[M+5H]^{5+}$; Mass found (ESI^+): 1491.9 $[M+2H]^{2+}$, 994.9 $[M+3H]^{3+}$, 746.7 $[M+4H]^{4+}$, 597.4 $[M+5H]^{5+}$. *UPLC Conditions:* C4 column at 40 °C using a 30-100% gradient of THF/formic acid (0.1%) in water/ formic acid (0.1%) over 4 minutes, then 100% THF/formic acid (0.1%) over 2 minutes.

^{1}H NMR (400 MHz, chloroform-d): δ_H 7.31-6.69 (aryl CH, 4H) 4.87-3.12 (NCH_2, 85H), 2.65 ((HC≡C)C<u>H</u>, 1H), 2.34-1.55 (acetate CH_3, CH_2, *i*-Bu CH & C≡CH, 19H), 1.38-1.22 (*t*-Bu CH_3, 108H) 0.93-0.85 (*i*-Bu CH_3, 42H);

^{31}P NMR (162 MHz, chloroform-d): δ_P 58.52;

HRMS (ES+): calculated for $C_{148}H_{259}N_{45}O_8P_6$ 2982.9770 $[M+H]^+$, found 2982.9790 $[M+H]^+$.

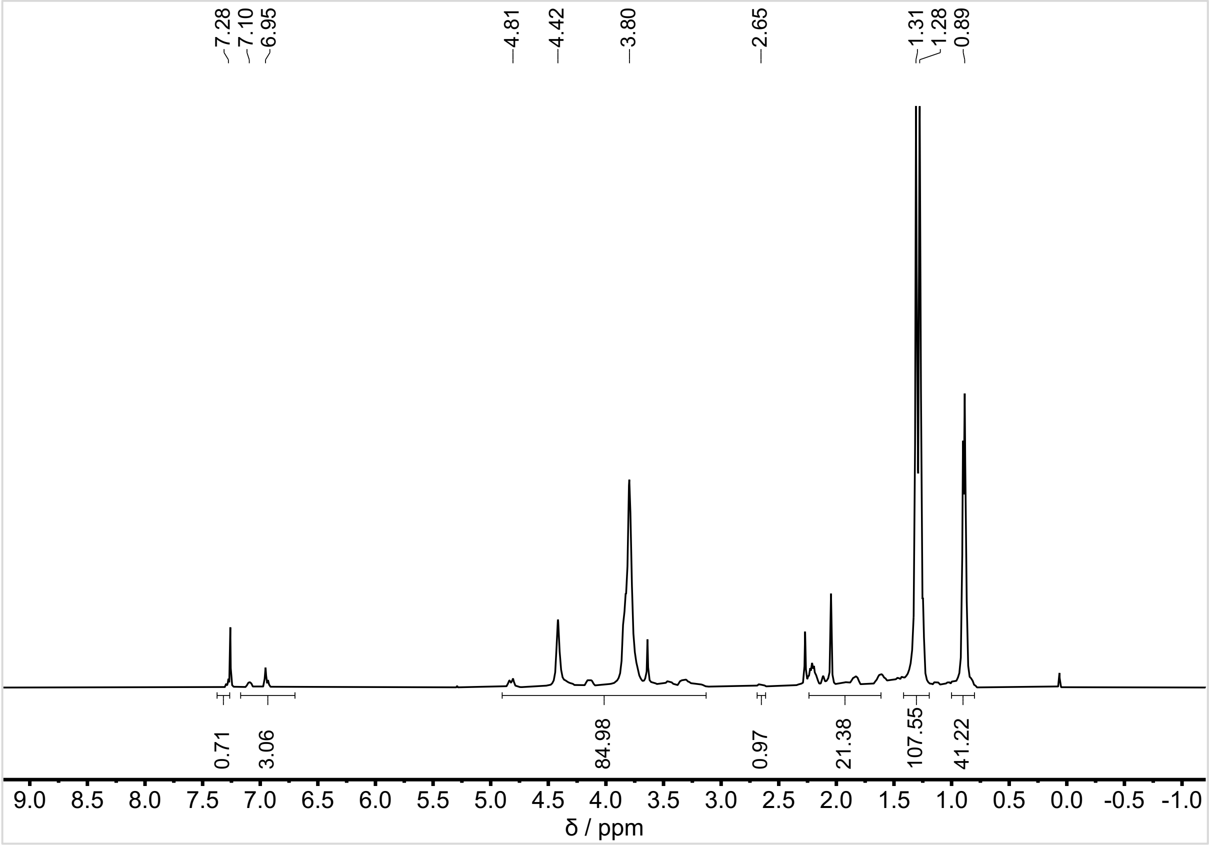

Figure S17: ^{1}H NMR (400 MHz, chloroform-d) spectrum of **zD*AAAAAAy**.

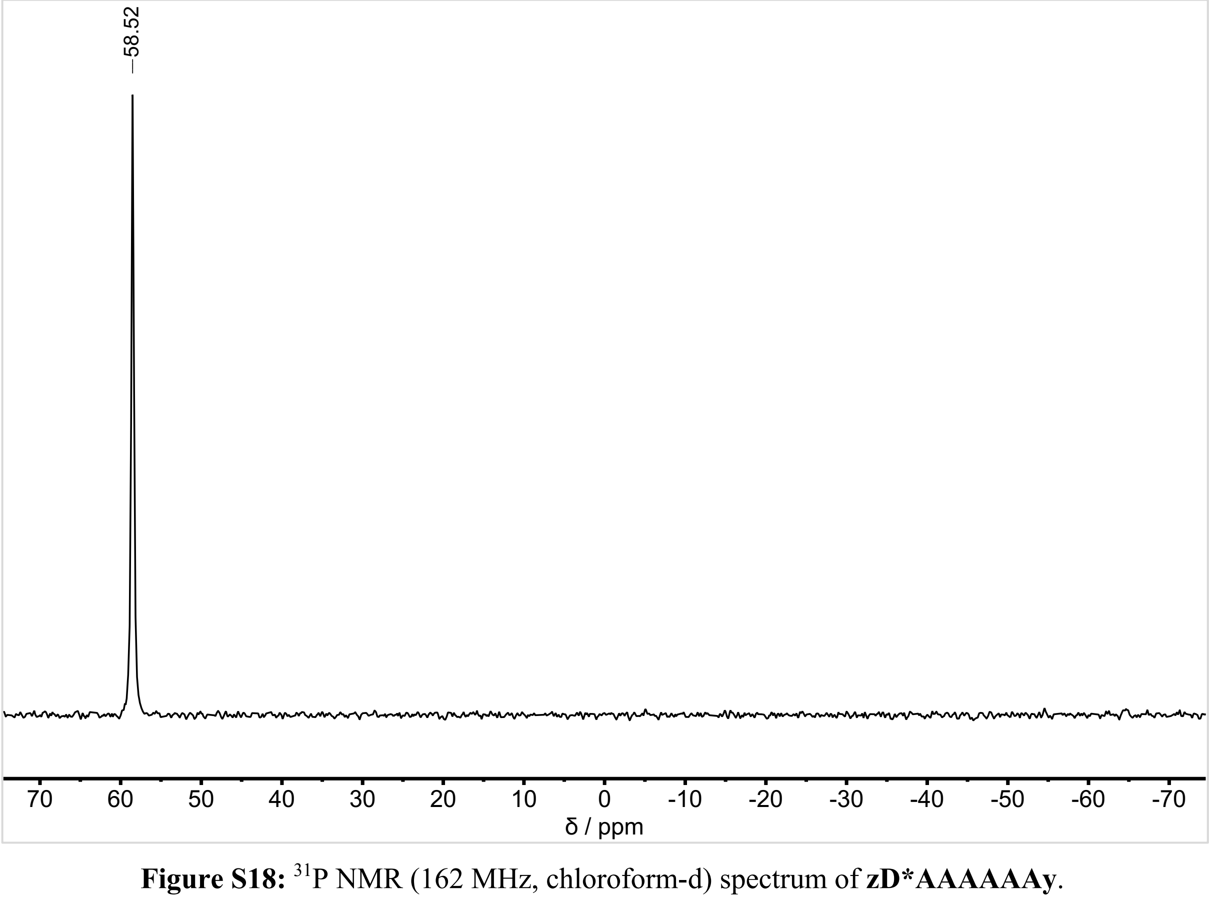

Figure S18: ^{31}P NMR (162 MHz, chloroform-d) spectrum of **zD*AAAAAAy**.

5. Characterisation of Oligomer Libraries

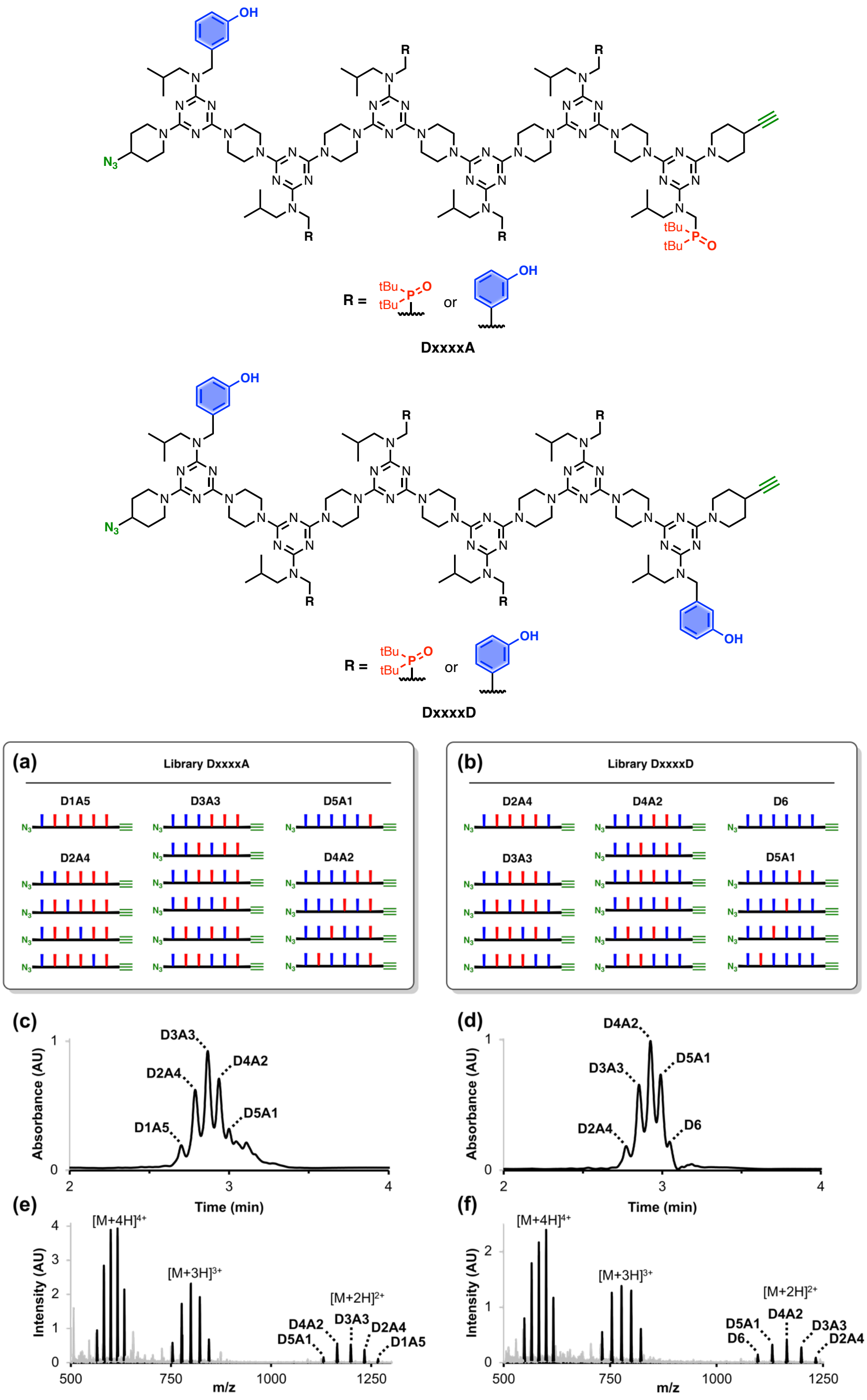

Figure S19: Schematic representation of the sequences present in **(a)** library **zDXXXXAy** and **(b)** library **zDXXXXDy**. UPLC traces of **(c)** library **zDXXXXAy** and **(d)** library **zDXXXXDy** with peaks labelled according to the composition of recognition units. ESI-MS of **(e)** library **zDXXXXAy** and **(f)** library **zDXXXXDy**. The $[M+2H]^{2+}$ peaks are labelled according to the composition of recognition units, and the corresponding $[M+3H]^{3+}$ and $[M+4H]^{4+}$ peaks are highlighted in black. *UPLC Conditions:* C4 column at 40 °C using a 30-100% gradient of THF/formic acid (0.1%) in water/formic acid (0.1%) over 4 minutes, then 100% THF/formic acid (0.1%) over 2 minutes.

	calculated m/z			found m/z		
	$[M+2H]^{2+}$	$[M+3H]^{3+}$	$[M+4H]^{4+}$	$[M+2H]^{2+}$	$[M+3H]^{3+}$	$[M+4H]^{4+}$
**D6**	1096.7	731.4	548.8	1096.7	731.3	548.7
**D5A1**	1130.7	754.1	565.9	1130.8	754.2	565.8
**D4A2**	1164.7	776.8	582.9	1164.7	776.7	582.8
**D3A3**	1198.7	799.5	599.9	1198.7	799.6	599.8
**D2A4**	1232.8	822.2	616.9	1232.8	822.2	616.8
**D1A5**	1266.8	844.9	633.9	1266.9	844.8	633.8

Table S1: Calculated and found ESI^+ masses for oligomer species with different numbers of donor and acceptor groups founds in libraries **zDXXXXAy** and **zDXXXXDy**.

Reactivity Difference Between Building Blocks

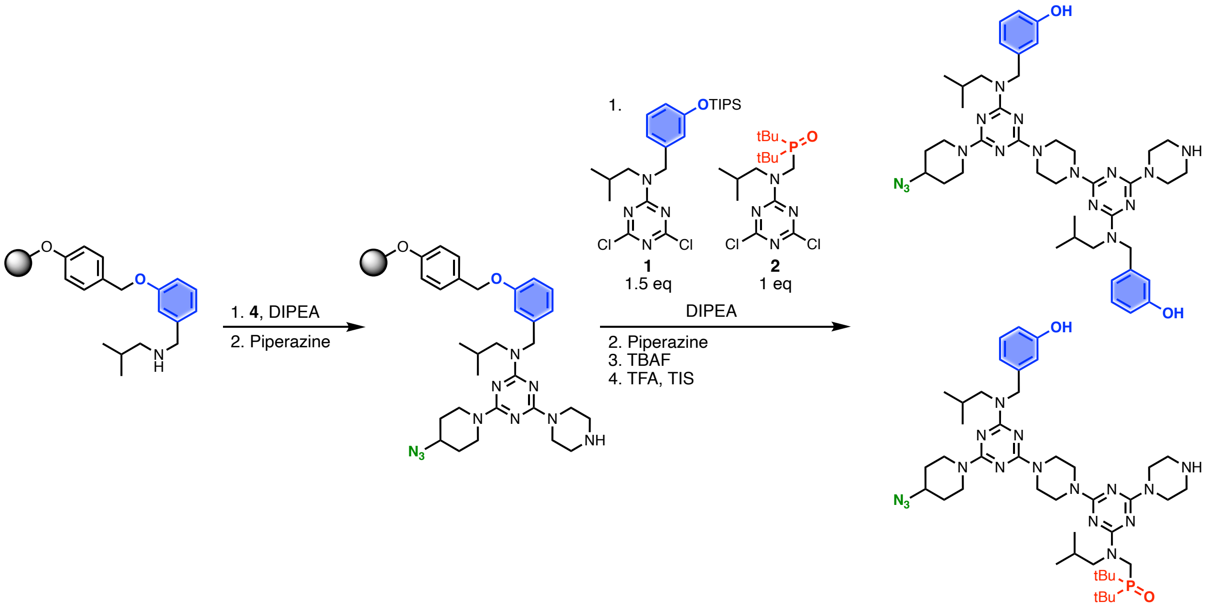

Scheme S1: SPS route to test the difference in reactivity between the phenol building block, **1**, and the phosphine oxide building block, **2**.

Scheme S1 shows the solid-phase synthesis route employing a 3:2 mixture of **1**:**2** in the second coupling cycle, to test whether this ratio accounted for the difference in rate of S_NAr reaction of the two building blocks. Equal amounts of the two final products are desired.

Figure shows the ^{1}H NMR spectrum at 373 K of the crude product mixture with the key peaks highlighted. Comparison of the integral for H_A with that of H_B and H_C shows that there is a 1:1 mixture of the two oligomers present, as desired. High temperature was required to resolve the peaks, since there are rotamers of the products which undergo slow interconversion at room temperature, giving rise to broad ^{1}H NMR signals.

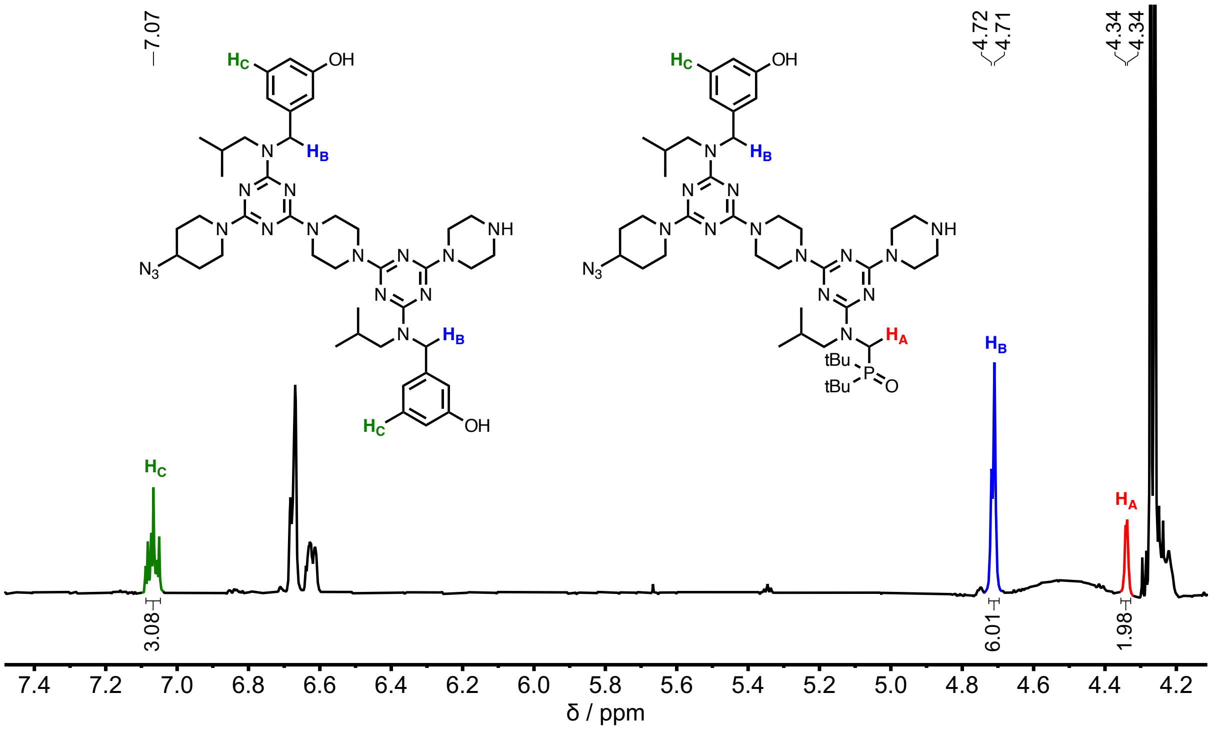

Figure S20: Downfield region of the ^{1}H NMR (500 MHz, DMSO-d$_6$) spectrum at 373 K of the mixture of the two products formed in Scheme S1.

6. NMR Experiments

NMR Titrations

Binding constants were measured by ^{31}P NMR or ^{1}H NMR titrations in a Bruker 400 MHz Avance III HD Smart Probe spectrometer. The host (m-cresol or **A**) was dissolved in TCE-d_2 at a known concentration. The guest (DMSO-d_6 or m-cresol) was dissolved in the host solution and made to known concentration. 0.6 mL of host was added to an NMR tube and the spectrum was recorded. Aliquots of guest in host solution were added to the NMR tube and the spectra were recorded after each addition. The chemical shifts of the host spectra were monitored as a function of guest concentration and analysed using Musketeer (available from the GitHub repository, https://github.com/daniilS/Musketeer/releases). The changes in chemical shift were fit to either a 1:1 binding isotherm, using equation 5, or a 1:2 binding isotherm. Errors are quoted as two standard deviations based on three different experiments.

The observed chemical shift (δ_{obs}, equation 1) is a weighted average of the chemical shifts of the free host (δ_H) and the host-guest complex (δ_{HG}):

$$\delta_{obs} = \delta_H \frac{[H]}{[H]_0} + \delta_{HG} \frac{[HG]}{[H]_0} \tag{1}$$

$$[H] = [H]_0 - [HG] \tag{2}$$

$$[G] = [G]_0 - [HG] \tag{3}$$

where $[H]_0$ is the initial concentration of host, $[G]_0$ is the initial concentration of guest, $[H]$ is the concentration of free host, $[G]$ is the concentration of free guest, and $[HG]$ is the concentration of host-guest complex.

Given that the association constant (K) for the 1:1 complex is $K = [HG]/[H][G]$, and substituting in equations 2 and 3, equation 1 can be re-written as:

$$\frac{\delta_{obs} - \delta_H}{\delta_{HG} - \delta_H} = \frac{[HG]}{[H]_0}$$

$$\frac{\delta_{obs} - \delta_H}{\delta_{HG} - \delta_H} = \frac{(K([H]_0 + [G]_0) + 1) \pm \sqrt{(K([H]_0 + [G]_0) + 1)^2 - 4K^2[H]_0[G]_0}}{2K[H]_0} \tag{4}$$

^{1}H NMR Titration of DMSO-zDDDDDDy into m-Cresol

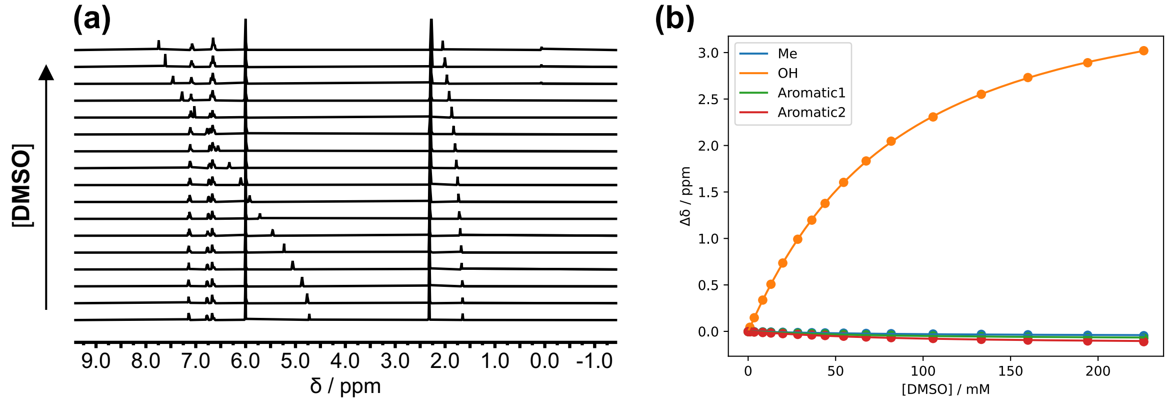

Figure S21: (a) ^{1}H NMR spectra (400 MHz) for the titration of DMSO-d_6 into m-cresol (30.6 mM in TCE-d_2, 298K). **(b)** Plot of the change in chemical shift of the ^{1}H NMR signal as a function of guest concentration, where the line represents the best fit to a 1:1 binding isotherm with $K = 17 \pm 1$ M^{-1}, $\delta_{free} = 4.72$ ppm and $\delta_{bound} = 8.65$ ppm (phenol OH signal).

^{31}P NMR Titration of m-Cresol into A Monomer (Compound 8)

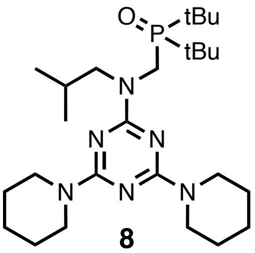

8

Compound **8** was synthesised according to the literature procedure found at:

Troselj, P., Bolgar, P., Ballester, P. & Hunter, C. A. High-Fidelity Sequence-Selective Duplex Formation by Recognition-Encoded Melamine Oligomers. *J. Am. Chem. Soc.* **143**, 8669–8678 (2021).

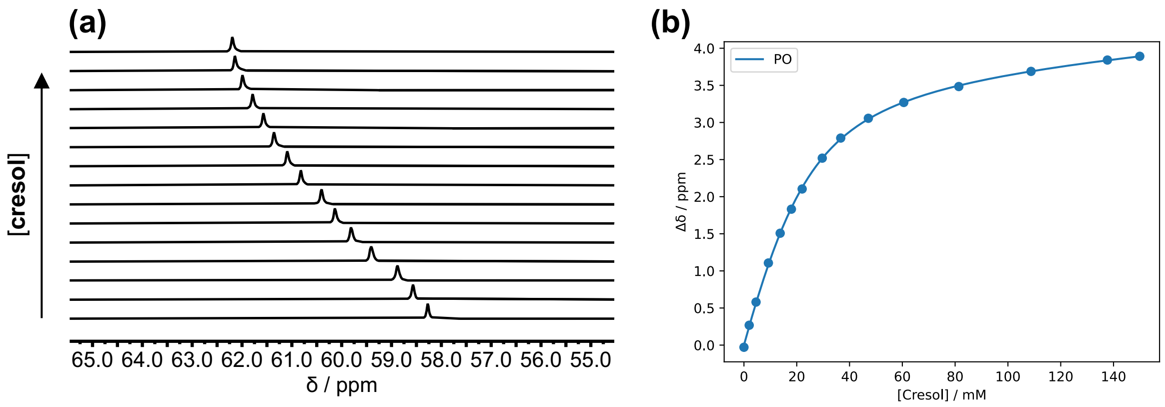

Figure S22: (a) ^{31}P NMR spectra (162 MHz) for the titration of m-cresol into **8** (20.8 mM in $CDCl_3$, 298K). **(b)** Plot of the change in chemical shift of the ^{31}P NMR signal as a function of guest concentration, where the line represents the best fit to a 1:2 binding isotherm with $K = 110 \pm 20$ M^{-1}, $\delta_{free} = 58.3$ ppm and $\delta_{bound} = 62.1$ ppm.

Thermal Denaturation of zD*AAAAAAy•zDDDDDDy Complex

Thermal denaturation data was measured by making a 1:1 solution of **zD*AAAAAAy** and **zDDDDDDy** oligomers at 1 mM concentration in tetrachloroethane-d_2 and measuring ^{31}P NMR spectra at different temperatures in a Bruker 500 MHz AVIII HD Smart Probe spectrometer equipped with a BCU Chiller unit. The temperature of the sample was changed using the internal thermostat of the NMR spectrometer, and the sample was allowed to equilibrate in the probe until the probe thermometer gave a stable temperature. The ^{31}P NMR chemical shifts of the acceptor homo-oligomer spectra were monitored as a function of temperature. Due to significant broadening of the ^{31}P NMR peaks, 50 Hz line broadening was applied to all spectra.

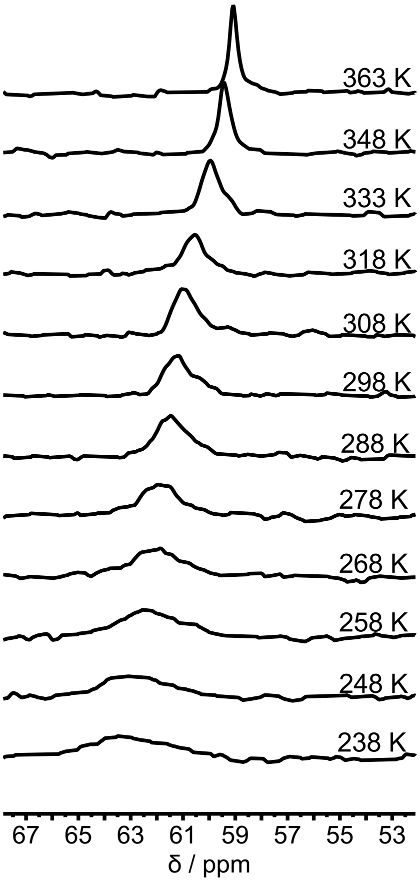

Figure S23: Variable temperature ^{31}P NMR spectra (202 MHz) for a 1 mM, 1:1 mixture of **zD*AAAAAAy** and **zDDDDDDy** in tetrachloroethane-d_2. Line broadening of 50 Hz was applied to all spectra.

DMSO Denaturation of zD*AAAAAAy•zDDDDDDy Complex

DMSO denaturation data was measured by ^{31}P NMR titrations on a Bruker 400 MHz Neo Prodigy spectrometer. To a 1:1 solution of **zD*AAAAAAy** and **zDDDDDDy** oligomers at 1 mM concentration in tetrachloroethane-d$_2$ (TCE-d$_2$) was added known volumes of DMSO-d$_6$ in TCE-d$_2$ and neat DMSO-d$_6$, and the spectrum recorded after each addition. The ^{31}P NMR chemical shifts of the acceptor homo-oligomer spectra were monitored as a function of DMSO-d$_6$ concentration (Figure S24b). Free ^{31}P NMR shifts were monitored for a 1 mM solution of **zD*AAAAAAy** in TCE-d$_2$ with the same addition concentrations of DMSO-d$_6$ to account for solvent effects (Figure S24a). The chemical shift values for titration of DMSO-d$_6$ into **zD*AAAAAAy** were subtracted from the chemical shift values for the denaturation experiment to account for solvent effects. The corrected data were analysed using using Musketeer (available from the GitHub repository, https://github.com/daniilS/Musketeer/releases).

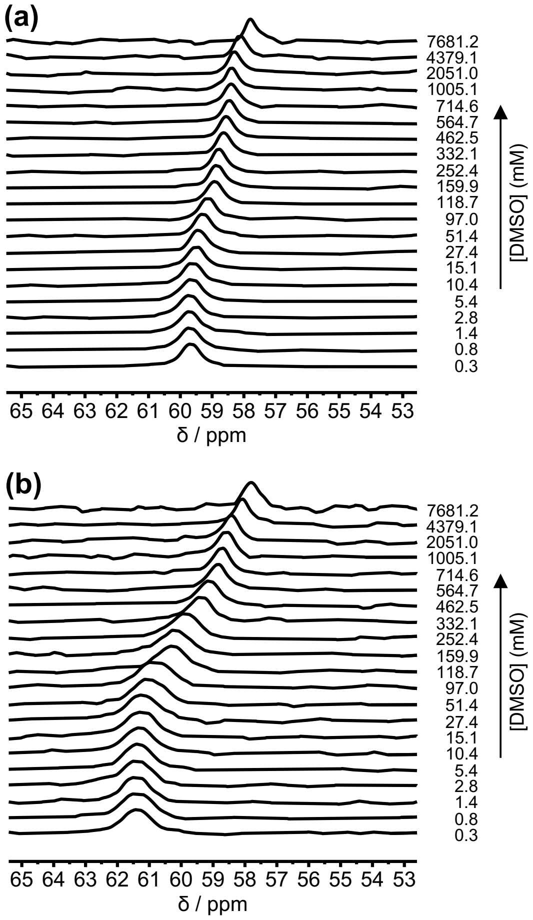

Figure S24: (a) ^{31}P NMR spectra (162 MHz) for titration of DMSO-d$_6$ into a 1 mM solution of **zD*AAAAAAy** in tetrachloroethane-d$_2$ at 298 K. **(b)** ^{31}P NMR spectra (162 MHz) for titration of DMSO-d$_6$ into a 1 mM, 1:1 mixture of **zDDDDDDy** and **zD*AAAAAAy** in tetrachloroethane-d$_2$ at 298 K. Line broadening of 50 Hz was applied to all spectra.

Figure S25 shows that the denaturation data does not fit well to a simple all or nothing isotherm that considers only duplex and single strands. We therefore implemented an isotherm that considers all possible intermediates (Figure S26).

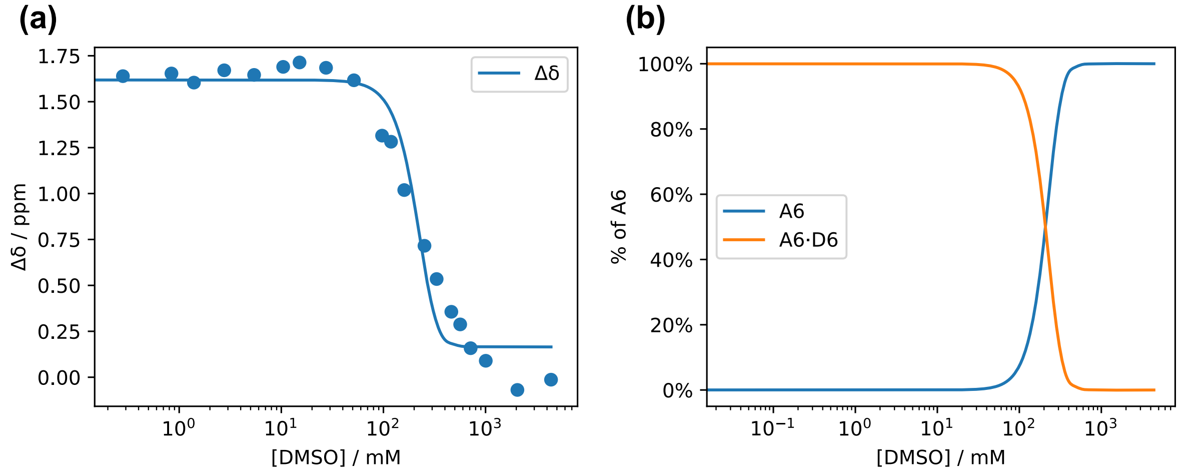

Figure S25: (a) Complexation-induced change in ^{31}P NMR chemical shift (Δδ) for **zD*AAAAAAy•zDDDDDDy** denaturation plotted as a function of DMSO-d_6 concentration in tetrachloroethane-d_2 at 298 K (Δδ is defined as the difference between the chemical shift of the 1:1 **zD*AAAAAAy•zDDDDDDy** mixture and pure **zD*AAAAAAy** at the same concentration of DMSO). The line was calculated using an all-or-nothing denaturation isotherm which only accounts for the fully bound duplex or the fully denatured complex. **(b)** Calculated speciation profile plotted as a function of DMSO-d_6 concentration in tetrachloroethane-d_2 at 298 K.

It was previously shown that the relationship between the association constant for duplex formation of REMOs (log K) and the number of base-pairs (N) was linear. Hence, the stepwise effective molarity (EM) for sequential formation of each intramolecular H-bond in the duplex can be assumed to be constant. The association constant for duplex formation between complementary oligomers of length N is given by equation 1:

$$K_N = 2{K_1}^N EM^{N-1} \tag{1}$$

where K_1 is the association constant for formation of a single intermolecular H-bond between a phenol and a phosphine oxide (110 ± 20 M^{-1} in TCE-d_2), and the statistical factor of 2 represents the degeneracy of the length N duplex compared to **A•D**.

The association constants of all of the partially denatured species can therefore be written as functions of K_1, K_d (the association constant for the complex formed between DMSO

and a **D** monomer) and EM. Isomeric arrangements are possible for most of the complexes, so statistical factors are included to account for degenerate species. K_d was measured by a ^{1}H NMR titration of DMSO into a solution of m-cresol in TCE-d$_2$ (K_d = 17 ± 1 M^{-1}). The value of EM is therefore only variable used to describe all of the association constants.

Figure S26 also shows that the chemical shifts of all of the partially denatured species can be written in terms of the chemical shift of a free and bound phosphine oxide (∂_{free} and ∂_{bound}). The observed chemical shift in the denaturation experiment can therefore be expressed as the population-weighted average of all of the species shown in Figure S26. The NMR data could therefore be fit to the isotherm described by Figure 26 by optimising just two variables, $\Delta\partial$ (∂_{bound} - ∂_{free}) and EM. The value of EM can then be used to obtain the association constants for all of the partially denatured species shown in Figure S26 and calculate the concentrations as a function of DMSO concentration (speciation plot in Figure 4c of the main text).

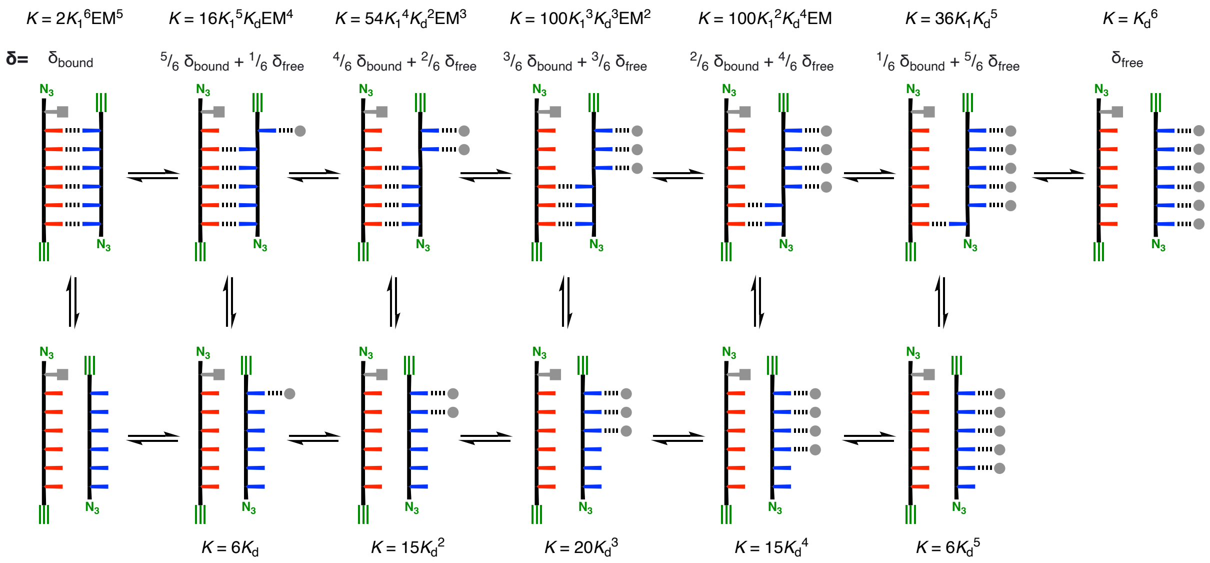

Figure S26: Equilibria involved in the DMSO (grey circles) denaturation of **zD*AAAAAAy•zDDDDDDy**. K_1 is the **A•D** association constant, K_d is the **D•**DMSO association constant, and EM is the effective molarity for formation of an intramolecular H-bond. δ_{bound} is the chemical shift of the fully bound duplex and δ_{free} is the chemical shift of the fully denatured species. There are degenerate isomeric arrangements of most of the complexes that give rise to statistical factors.

7. Duplex Trapping Experiments

General Procedure for CuAAC Trapping Experiments

For each experiment, fresh stock solutions of oligomers, 4-*t*-butylbenzyl azide and $Cu(MeCN)_4PF_6$-TBTA in dry DCM were prepared. The calculated amount from stock solutions of oligomers and 4-*t*-butylbenzyl azide were transferred to a reaction vial and the solvent was removed via evaporation under a stream of N_2. Cu(I)-TBTA in dry DCM was then added to the reaction vial under a stream of N_2, and mixtures were left to stir for 48 hours at room temperature. After reaction completion, the DCM was evaporated under a stream of N_2 and mixtures were re-dissolved in THF/MeOH to a known concentration, then sonicated to ensure that the products were fully dissolved. Mixtures were injected into the LCMS (between 2-5 μL injections) and UV peaks were identified (λ 254 nm). Peak identity was assigned based on the corresponding mass spectra and retention time. One UPLC gradient was used for analysis of product mixtures.

A_6 CuAAC Trapping Control Experiments

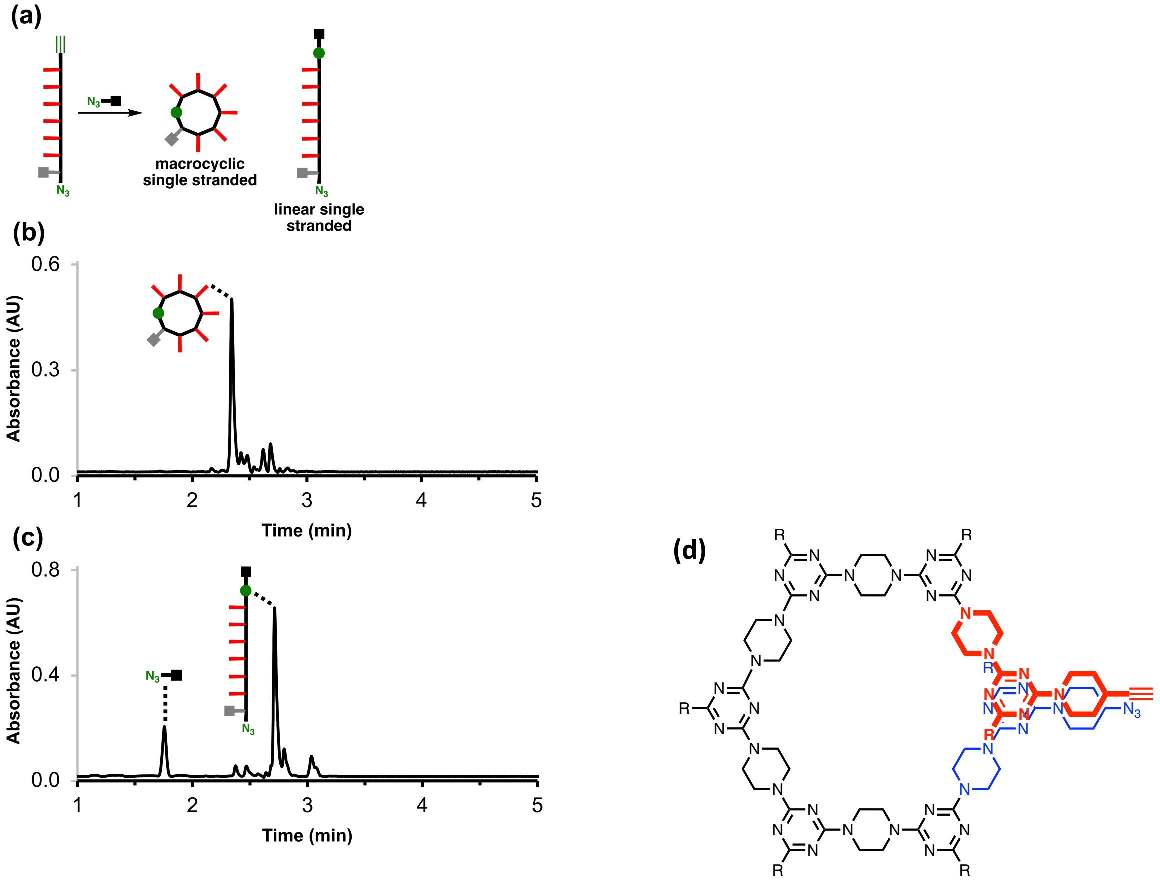

Figure S27: (a) Schematic representation of products formed after CuAAC reaction of **zD*AAAAAAy** in the presence of a competing azide (green circles represent triazoles in the products). **(b)** UPLC trace after reaction of **zD*AAAAAAy** (50 µM), 4-*t*-butylbenzyl azide (100 µM) and Cu(MeCN)$_4$PF$_6$-TBTA (0.4 mM) in dichloromethane at room temperature for 48 hours. **(b)** UPLC trace after reaction of **zD*AAAAAAy** (50 µM), 4-*t*-butylbenzyl azide (5 mM) and Cu(MeCN)$_4$PF$_6$-TBTA (0.4 mM) in dichloromethane at room temperature for 48 hours. (d) Folded conformation of **zD*AAAAAAy** that brings the red and blue end groups into close proximity. *UPLC Conditions:* C4 column at 40 °C using a 30-100% gradient of THF/formic acid (0.1%) in water/formic acid (0.1%) over 4 minutes, then 100% THF/formic acid (0.1%) over 2 minutes.

Hexamer Library CuAAC Trapping

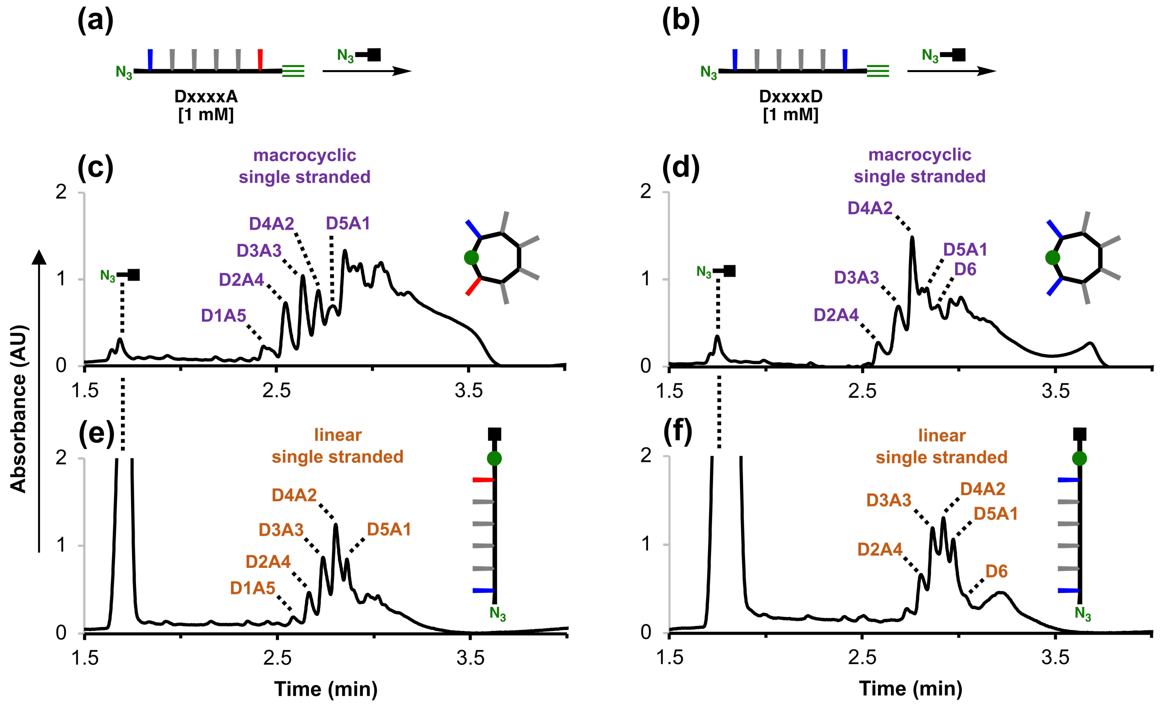

Figure S28: (a) Schematic representation of the CuAAC reaction of library **zDXXXXAy** in the presence of a competing azide. **(b)** Schematic representation of the CuAAC reaction of library **zDXXXXDy** in the presence of a competing azide. **(c)** UPLC trace after reaction of library **zDXXXXAy** (1 mM), 4-*t*-butylbenzyl azide (1 mM) and Cu(MeCN)$_4$PF$_6$-TBTA (4 mM) in dichloromethane at room temperature for 48 hours. **(d)** UPLC trace after reaction of library **zDXXXXDy** (1 mM), 4-*t*-butylbenzyl azide (1 mM) and Cu(MeCN)$_4$PF$_6$-TBTA (4 mM) in dichloromethane at room temperature for 48 hours. **(e)** UPLC trace after reaction of library **zDXXXXAy** (1 mM), 4-*t*-butylbenzyl azide (50 mM) and Cu(MeCN)$_4$PF$_6$-TBTA (4 mM) in in dichloromethane at room temperature for 48 hours. **(f)** UPLC trace after reaction of library **zDXXXXDy** (1 mM), 4-*t*-butylbenzyl azide (50 mM) and Cu(MeCN)$_4$PF$_6$-TBTA (4 mM) in dichloromethane at room temperature for 48 hours. Green circles represent triazoles in the products. *UPLC Conditions:* C4 column at 40 °C using a 30-100% gradient of THF/formic acid (0.1%) in water/formic acid (0.1%) over 4 minutes, then 100% THF/formic acid (0.1%) over 2 minutes.

Identification of Macrocyclic Duplex Products using EIC

Control experiment on the zDDDDDDy•zD*AAAAAAy duplex

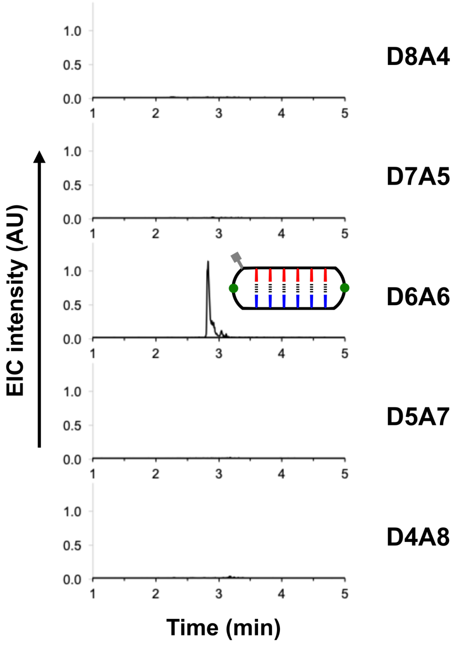

Figure S29: Extracted ion chromatograms (EIC) for the $[M+5H]^{5+}$ ions of macrocyclic duplexes with different numbers of recognition units for the products of the CuAAC reaction of **zDDDDDDy** (50 μM), **zD*AAAAAAy** (50 μM), 4-*t*-butylbenzyl azide (100 μM) and $Cu(MeCN)_4PF_6$-TBTA (0.4 mM) in dichloromethane at room temperature for 48 hours. *UPLC Conditions:* C4 column at 40 °C using a 30-100% gradient of THF/formic acid (0.1%) in water/formic acid (0.1%) over 4 minutes, then 100% THF/formic acid (0.1%) over 2 minutes.

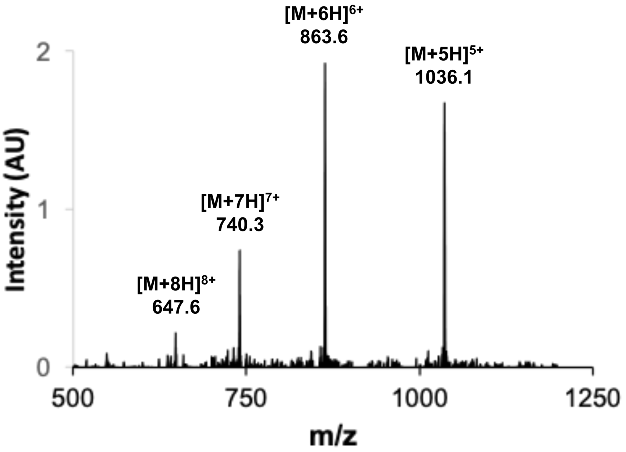

Figure S30: ESI-MS of the EIC peak corresponding to the **D6A6** macrocycle duplex in Figure S28. Calculated mass: 1035.9 $[M+5H]^{5+}$, 863.4 $[M+6H]^{6+}$, 740.2 $[M+7H]^{7+}$, 647.8 $[M+8H]^{8+}$; Mass found (ESI^+): 1036.1 $[M+5H]^{5+}$, 863.6 $[M+6H]^{6+}$, 740.3 $[M+7H]^{7+}$, 647.6 $[M+8H]^{8+}$.

Identification of duplexes in the hexamer libraries

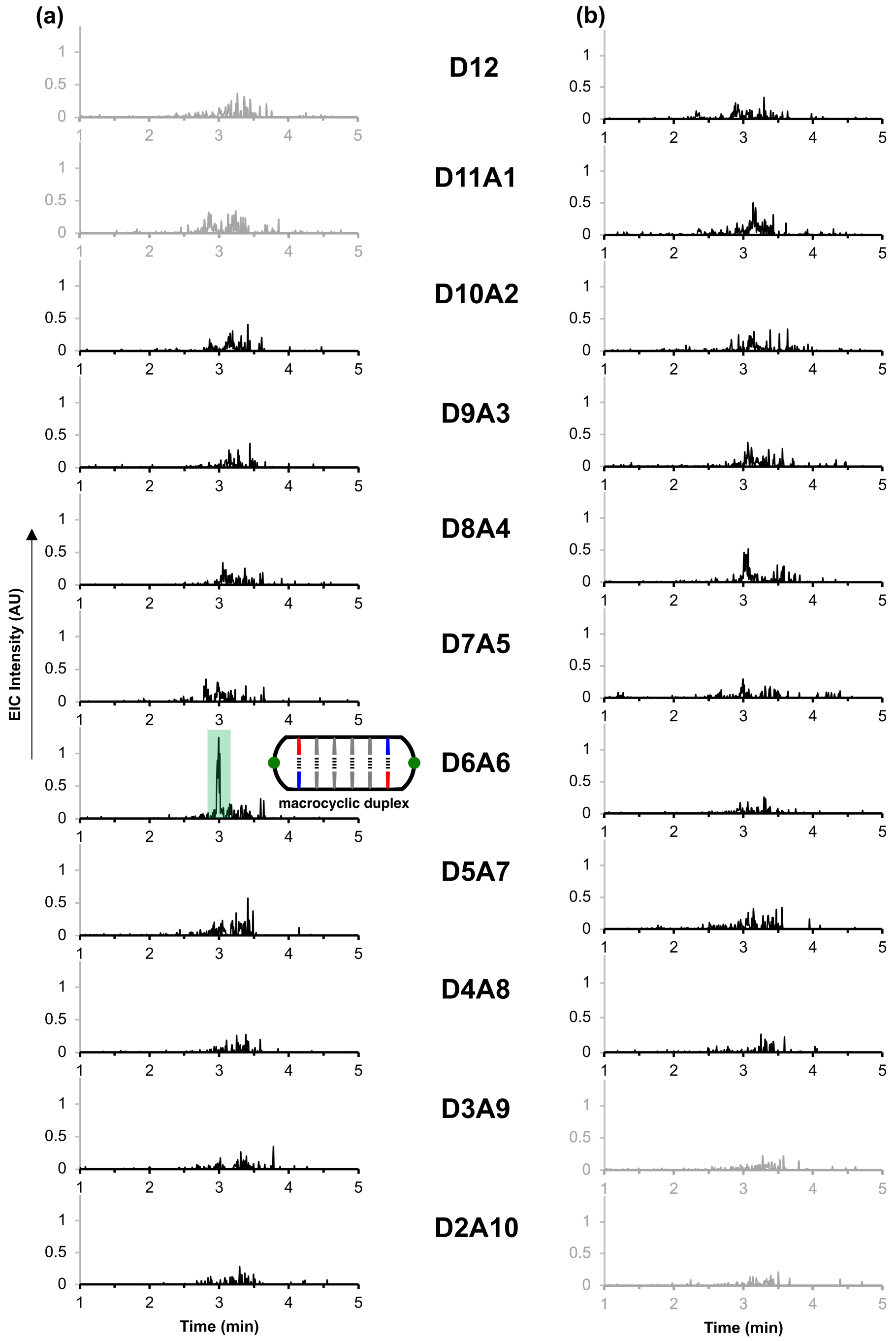

Figure S31: Extracted ion chromatograms (EIC) for the $[M+5H]^{5+}$ ions of macrocyclic duplexes with different numbers of recognition units. **(a)** Products of CuAAC reaction of library **zDXXXXAy**; **(b)** Products of CuAAC reaction of library **zDXXXXDy**. Reactions were carried out using 1 mM concentrations of the library, 4-*t*-butylbenzyl azide (1 mM) and $Cu(MeCN)_4PF_6$-TBTA (0.4 mM) in dichloromethane at room temperature for 48 hours. *UPLC Conditions:* C4 column at 40 °C using a 30-100% gradient of THF/formic acid (0.1%) in water/formic acid (0.1%) over 4 minutes, then 100% THF/formic acid (0.1%) over 2 minutes.

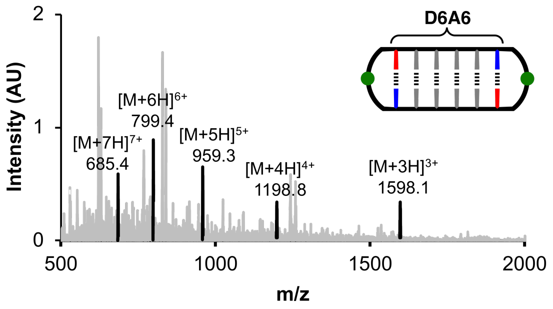

Figure S32: ESI-MS of the EIC peak corresponding to the **D6A6** macrocycle duplex in Figure . The peaks in grey correspond to linear single stranded species with the same retention time as **D6A6**. Calculated mass: 1598.0 $[M+3H]^{3+}$, 1198.8 $[M+4H]^{4+}$, 959.2 $[M+5H]^{5+}$, 799.5 $[M+6H]^{6+}$, 685.4 $[M+7H]^{7+}$; Mass found (ESI^{+}): 1598.1 $[M+3H]^{3+}$, 1198.8 $[M+4H]^{4+}$, 959.3 $[M+5H]^{5+}$, 799.4 $[M+6H]^{6+}$, 685.4 $[M+7H]^{7+}$.
